# Collective analysis of the expression and prognosis for LEM-domain proteins in prostate cancer

**DOI:** 10.1186/s12957-022-02640-z

**Published:** 2022-06-02

**Authors:** Tianzhen He, Yulian Zhang, Xueyu Li, Caihong Liu, Guanqun Zhu, Xinbao Yin, Zongliang Zhang, Kai Zhao, Zhenlin Wang, Peng Zhao, Ke Wang

**Affiliations:** 1 Institute of Special Environmental Medicine, Nantong University, Nantong, 226019 China; 2 Department of Gynecology, The Affiliated Hospital of Qingdao University, Qingdao University, No. 16 Jiangsu Road, Shinan District, Qingdao, 266000 Shandong Province China; 3 Department of Urology, The Affiliated Hospital of Qingdao University, Qingdao University, No. 16 Jiangsu Road, Shinan District, Qingdao, 266000 Shandong Province China; 4 Western Administrative Office Center, Qingdao West Coast New District Health Bureau, No. 166 Shuangzhu Road, Huangdao District, Qingdao, 266000 Shandong Province China; 5 Faculty of Sport Science and Coaching, Universiti Pendidikan Sultan Idris, 35900 Tanjong Malim, Perak Darul Ridzuan Malaysia; 6 Athletics Department, Duke Kunshan University, Kunshan, 215316 Jiangsu Province China

**Keywords:** LEM-domain proteins, Prostate adenocarcinoma, Immune infiltration, Survival prognosis, Gene mutations

## Abstract

**Background:**

Mammalian LEM-domain proteins (LEMs) are encoded by seven genes, including LAP2, EMD, LEMD1, LEMD2, LEMD3, ANKLE1, and ANKLE2. Though some LEMs were involved in various tumor progression, the expression and prognostic values of LEMs in prostate adenocarcinoma (PRAD) have yet to be analyzed.

**Methods:**

Herein, we investigated the expression, survival data, and immune infiltration levels of LEMs in PRAD patients from ATCG, TIMER, LinkedOmics, and TISIDB databases. We also further validated the mRNA and protein expression levels of ANKLE1, EMD, and LEMD2 in human prostate tumor specimens by qPCR, WB, and IHC.

**Results:**

We found that all LEM expressions, except for that of LAP2, were markedly altered in PRAD compared to the normal samples. Among all LEMs, only the expressions of ANKLE1, EMD, and LEMD2 were correlated with advanced tumor stage and survival prognosis in PRAD. Consistent with the predicted computational results, the mRNA and protein expression levels of these genes were markedly increased in the PRAD group. We then found that ANKLE1, EMD, and LEMD2 expressions were markedly correlated with immune cell infiltration levels. High ANKLE1, EMD, and LEMD2 expressions predicted a worse prognosis in PRAD based on immune cells. DNA methylation or/and copy number variations may contribute to the abnormal upregulation of ANKLE1, EMD, and LEMD2 in PRAD.

**Conclusions:**

Taken together, this study implied that ANKLE1, EMD, and LEMD2 were promising prognosis predictors and potential immunotherapy targets for PRAD patients.

**Supplementary Information:**

The online version contains supplementary material available at 10.1186/s12957-022-02640-z.

## Background

LEM-domain proteins (LEMs), named for the founding human members Lamina-associated polypeptide 2 (LAP2, or designated as TMPO), Emerin (EMD), and MAN1 (also known as LEMD3), represent one family of lamin-interacting proteins. In mammalian genomes, seven individual genes encode LEMs, including ANKLE1, ANKLE2, LEMD1, LEMD2, LAP2, EMD, and MAN1, five of which encode inner nuclear membrane proteins. Of those, ANKLE1 was an endonuclease possibly involved in DNA repair [1]. ANKLE2 was a regulator of BAF phosphorylation with a role in post-mitotic nuclear envelope formation [2, 3]. LEMD1 was associated with testis-predominant transcription [4]. LEMD2 was required for nuclear integrity [5]. EMD mediated membrane anchorage to the cytoskeleton and interacted with barrier-to-autointegration factor (BAF) and lamins [6]. The mammalian LAP2 gene, also known as thymopoietin (TMPO), encodes six splice isoforms (α, β, γ, δ, ε, ζ) regulating the nuclear architecture by binding lamin B1 and chromosomes [7]. LAP2, EMERIN, and MAN1 interacted with the BAF and, hence, indirectly interact with the chromatin [8–10]. Thus, the LEM family members may play an important role in the cell cycle. Furthermore, the expressions of LAP2 and LEMD1 were upregulated in various digestive tract cancers (stomach, liver, pancreas, and bile duct) [11], colorectal cancer [4], lymphoma cells [12], and prostate cancer [13].

Prostate cancer is the second most frequent cancer diagnosis made in men and the fifth leading cause of death worldwide, with 1,276,106 new cases and 358,989 deaths according to GLOBOCAN 2018 estimates [14]. The primary therapeutic methods for PRAD are surgery, chemotherapy, radiation therapy, and androgen deprivation therapy, markedly improving the therapeutic effects. There is growing documentation that these therapy strategies have profound effects on the immune system [15–18]. Immunotherapy is becoming a new attractive treatment option. The immune checkpoint inhibitor PD-L1 was shown to be associated with clinical progression in PRAD [19]. Notably, targeting sipuleucel-T, CTLA-4, PD-1, and PD-L1 has been approved or is being explored as prostate cancer treatments [20]. Due to tumor heterogeneity, exploring new potential immunotherapy targets for effectively enhancing prognosis and individualized treatment is necessary.

The tumor microenvironment contains infiltrating immune cells, stromal cells, extracellular matrix, and tumor cells. Studies showed that tumor-infiltrating immune cells have different roles in the development of tumors. For example, N1 tumor-associated neutrophils suppressed tumor growth. In contrast, N2 tumor-associated neutrophils were shown to induce tumorigenesis and immunosuppression [21]. For LEMs, ANKLE1 may control tumor development via DNA damage and repair process [22, 23]. Depletion of EMD promoted metastasis by inducing nuclear shape instability [24]. Hence, the LEMs have multifaceted functions in the tumor microenvironment. However, the underlying mechanisms of LEMs in PRAD progression and tumor-infiltrating lymphocytes remain unclear.

This study used the LinkedOmics database and R language to analyze LEM expressions and their association with the prognosis. Furthermore, we used TISIDB, TIMER, and UCSC Xena databases to investigate the correlation between ANKLE1, EMD, and LEMD2 expressions and tumor-infiltrated immune cells in the tumor microenvironment. We also further explored the molecular mechanisms of ANKLE1, EMD, and LEMD2 dysregulation, such as analysis of the DNA methylation, somatic mutations, and copy number variations. Our findings unveiled the crucial role of ANKLE1, EMD, and LEMD2 in PRAD prognosis. We further provided an underlying mechanism of the expressions of ANKLE1, EMD, and LEMD2 in potentially regulating the infiltration levels of immune cells, partly affecting the prognosis of PRAD.

## Methods

### Data acquisition and processing

The RNA-seq level 3 data and clinical data of PRAD patients were downloaded from the TCGA database (https://portal.gdc.cancer.gov/) and included 499 tumor samples and 52 normal samples. We used the deseq2 package (R version 3.6.3) for differentially expressed genes and clinical relevance analyses and ggplot2 for data visualization [25]. The Wilcoxon rank-sum test was applied to assess the differential expressions of LEM-domain proteins.

### KM survival and time-dependent survival ROC analysis

Survival analysis is a set of methods for evaluating time-to-event data that is widely applied across research disciplines. Standard survival analysis used the TCGA PRAD data and utilized the survminer and survival packages to display the KM plots [26]. We calculated hazard ratios (HRs) of 95% confidence intervals (CIs) and the log-rank *p*-value. The time-dependent curve of diagnosis was created using the “timeROC” R package (version 0.4). The TIMER web server (https://cistrome.shinyapps.io/timer/) was used to explore the relationship between immune-infiltrating cells/immune cell markers and gene expression that affects the clinical prognosis in PRAD. The levels of the gene expression were expressed as log2 RSEM.

### LinkedOmics database analysis

The LinkedOmics database (http://www.linkedomics.org/login.php) was used to analyze 32 TCGA cancer-associated multidimensional datasets. The co-expressed genes associated with LEM-domain proteins (including ANKLE1, EMD, and LEMD2) were identified from the TCGA PRAD cohort through the LinkFinder module in the database, and the correlation of results was tested by the Pearson correlation coefficient and showed respectively in volcano plot and heat maps. Function module analyses of the Gene Ontology biological process (GO_BP), Gene Ontology cellular component (GO_CC), Gene Ontology molecular function (GO_MF), and Kyoto Encyclopedia of Genes and Genomes (KEGG) pathways were analyzed by the gene set enrichment analysis (GSEA) in the LinkInterpreter module.

### Assessment of immune cell infiltration

The GSVA package (R version 3.6.3) was used to infer the abundance of tumor-infiltrating immune cells from the gene expression profiles of the PRAD samples in the TCGA dataset [27]. In this study, twenty-four immune cell phenotypes were analyzed, including activated DC (aDC), B cells, CD8 T cells, cytotoxic cells, DC, eosinophils, immature DC (iDC), macrophages, mast cells, neutrophils, NK CD56^bright^ cells, NK CD56^dim^ cells, NK cells, plasmacytoid DC (pDC), T cells, T helper cells, T central memory (Tcm), T effector memory (Tem), T follicular helper (Tfh), T gamma delta (Tgd), Th1 cells, Th17 cells, Th2 cells, and Treg cells [28]. We used Spearman’s correlation analysis to evaluate the correlation of gene expression and immune infiltration cells.

### TISIDB database analysis

The TISIDB database (http://cis.hku.hk/TISIDB) was utilized to analyze the associations of LEM-domain proteins (including ANKLE1, EMD, and LEMD2) with lymphocytes, immunomodulators, and chemokines (or receptors).

### UCSC Xena database

UCSC Xena database (http://xena.ucsc.edu/) is a genome-related database, which brings approximately 200 public databases together, including TCGA, ICGC, TARGET, GTEx, and CCL. The database was used to examine the copy number and DNA methylation, somatic mutation, gene expression, and protein expression. Pearson’s correlation coefficient was used to evaluate the association between DNA methylation, gene mutation, and CNV and mRNA expressions of ANKLE1, EMD, and LEMD2.

### Human prostate tumor sample collection

Fresh human prostate tumor specimens were collected and divided into two parts: one part was sent to pathology for examination, and the other was kept for qPCR, WB, and IHC. After confirmation by pathological examination, these human prostate tumor specimens were employed to determine the mRNA and protein expression levels of ANKLE1, EMD, and LEMD2 by qPCR, WB, and IHC. All prostate cancer specimens used in our study were Gleason score 9.

### qRT-PCR

Total RNA extraction was performed according to the manufacturer’s protocol (Takara RNAiso Plus Kit). Then, qRT-PCR was performed using a AG Biotechnology RT-PCR Kit with SYBR Green and specific primers. GAPDH-specific primers served as internal controls (Sangon Biotech, B661104). qRT-PCR primer sequences were shown as follows: human ANKLE1 forward primer GACCCCAACGCTCGATCTG, reverse primer TCGGGCTCCTGAGTCTCTG; human EMD forward primer GCCATGGACAACTACGCAGA, reverse primer GTCGTCATTGTAGCCCTTGC; and human LEMD2 forward primer GGCTTGGTAATGCTTTACTCCC, reverse primer CTTGGCCTGACAGAACTCAT.

### Immunohistochemistry

Three-millimeter tumor sections were incubated with commercial polyclonal antibodies against ANKLE1 (Proteintech, 24080-1-AP), EMD (Proteintech, 10351-1-AP), and LEMD2 (Boster, M00714) at 1:200 dilution overnight at 4 °C. Then, the sections were conjugated with horseradish peroxidase (HRP) antibody (1:200 dilution; Servicebio) at room temperature for 2 h, then covered by 3, 3-diaminobenzidine (DAB) (Servicebio, G1211). Then, all fields were observed under light microscopy. Control experiments without primary antibodies demonstrated that the signals observed were specific.

### Western blot

For protein expression levels of ANKLE1, EMD, and LEMD2, the cell lysates were prepared by using RIPA (Elabscience). The above samples were subjected to SDS-PAGE gel, and the separated proteins were transferred onto the PVDF membranes. After blocking with 5% non-fat milk, the membranes were probed with primary Abs ANKLE1 (Proteintech, 24080-1-AP, 1:1000), EMD (Proteintech, 10351-1-AP, 1:1000), and LEMD2 (Boster, M00714, 1:6000) overnight at 4 °C and then incubated with HRP-conjugated secondary Abs (1:7000) at room temperature for 2 h. The protein bands were examined by Fusion Fx7 (VILBER LOURMAT).

### Statistical analysis

The LEM expressions were analyzed via the R project (3.6.3 version). Survival curves were generated using the TIMER web server and R project using the “survival” package. Spearman’s correlation analysis was used to evaluate the correlation of the gene expression in the TIMER. For bioassay validation, comparisons of the two groups of data were analyzed by a two-tailed Student’s *t*-test by using GraphPad Prism 7.0. (GraphPad, San Diego, CA). All *p* values less than 0.05 were considered significant.

## Results

### Expression levels of LEM-domain proteins in patients with prostate cancer

**Fig. 1:**
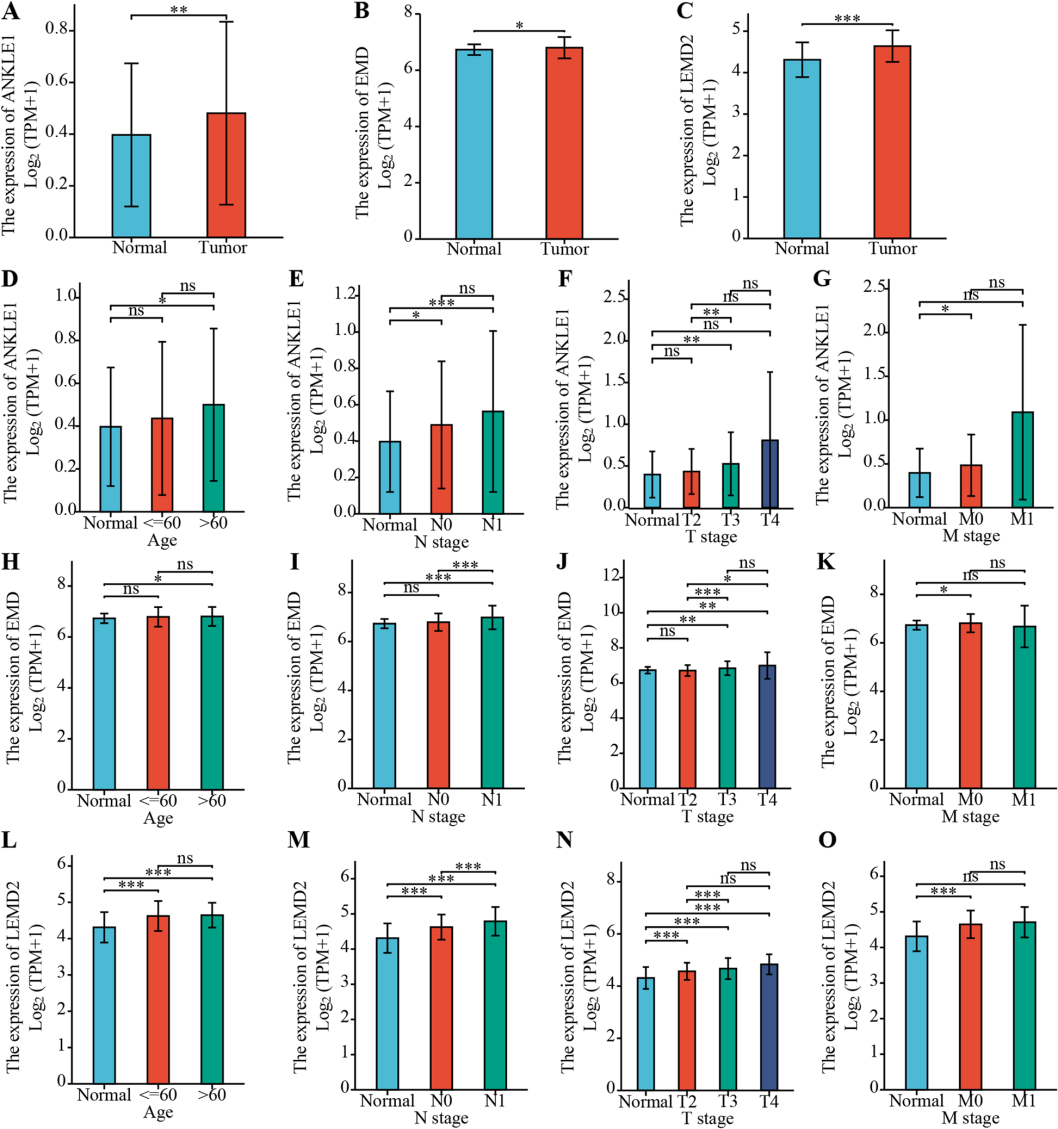
The expressions of ANKLE1, EMD, and LEMD2 and their relationship with clinical parameters of PRAD. **A**–**C** ANKLE1, EMD, and LEMD2 levels were increased in prostate cancer tissues compared to normal tissues (RNA-seq data from TCGA PRAD). The number of the normal group is 52, and the number of the tumor group is 499. **D**–**G** Higher ANKLE1 expression was associated with age, N stage, T stage, and M stage. **H**–**K** Higher EMD expression was associated with age, N stage, T stage, and M stage. **L**–**O** Higher LEMD2 expression was associated with age, N stage, T stage, and M stage. Compared with indicated group, *p < 0.05, **p < 0.01, ***p < 0.001. n.s., no significant difference

Seven LEM-domain proteins have been identified in mammalian cells. We compared the expression levels of LEM-domain proteins in PRAD with those in normal samples of the TCGA database by using the R language (Fig. 1A–C and Additional file 1: Fig. S1). The results showed that ANKLE1, EMD, and LEMD2 were highly expressed in PRAD (Fig. 1A–C). Then, the mRNA levels of ANKLE1, EMD, and LEMD2 in the TIMER database were determined, and these gene expression levels were also upregulated in PRAD (Additional file 1: Fig. S1A-S1C). The expression of LEMD1 was significantly downregulated in PRAD, compared to normal samples (Additional file 1: Fig. S1D, *p* <0.001). However, the expressions of ANKLE2, TMPO, and LEMD3 had no significant differences between PRAD and normal samples (Additional file 1: Fig. S1E-S1G, n.s.: no significant difference). In addition, the expressions of ANKLE1, EMD, and LEMD2 were associated with clinical parameters of PRAD, including age, N stage, T stage, and M stage (Fig. 1D–O).

### Prognostic significance of qPCR validation of ANKLE1, EMD, and LEMD2 in PRAD

**Fig. 2:**
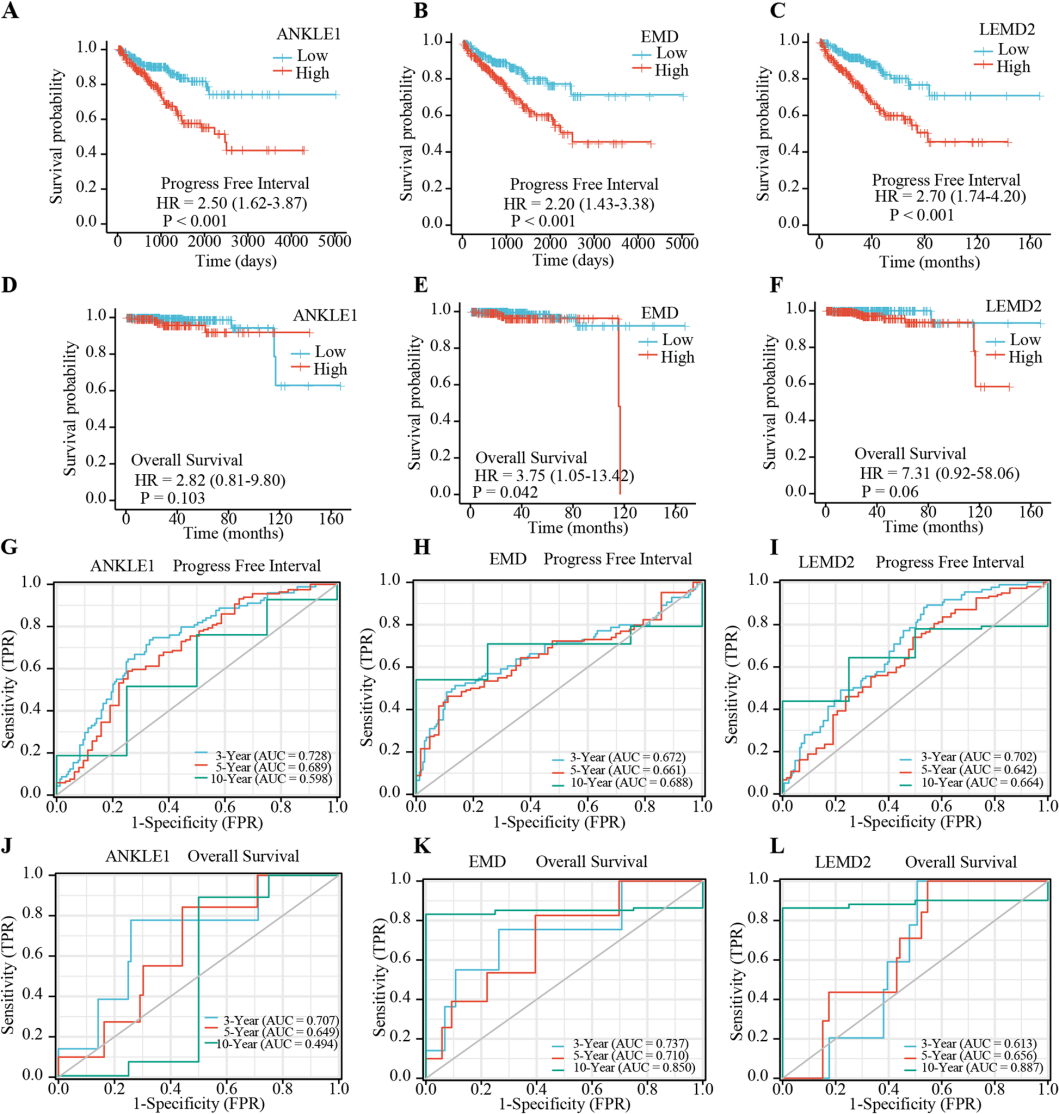
The prognostic analysis of ANKLE1, EMD, and LEMD2 in prostate cancer. **A**–**C** The progress-free interval of ANKLE1, EMD, and LEMD2 mRNA level in prostate cancer patients (Kaplan-Meier plotter, tumor samples: *n* =499). **D**–**F** The overall survival of ANKLE1, EMD, and LEMD2 mRNA level in prostate cancer patients (Kaplan-Meier plotter, tumor samples: *n* = 499). **G**–**L** Time-dependent survival ROC curve analysis of ANKLE1, EMD, and LEMD2 to predict 3-, 5-, and 10-year survival rates

We investigated the Kaplan-Meier plotter for the prognostic significance of these seven LEM-domain proteins by using the survminer and survival packages of the R project. As a result, only ANKLE1, EMD, and LEMD2 were associated with the prognostic significance of PRAD. High levels of ANKLE1, EMD, and LEMD2 predicted poor prognosis in PRAD (Fig. 2A–F). Time-dependent survival ROC curves of ANKLE1, EMD, and LEMD2 were further created to predict 3-, 5-, and 10-year survival rates. All these AUC values of 3- and 5-year survival rates were above 0.6, which is considered suitable for the prediction in PRAD (Fig. 2G–L).

**Fig. 3:**
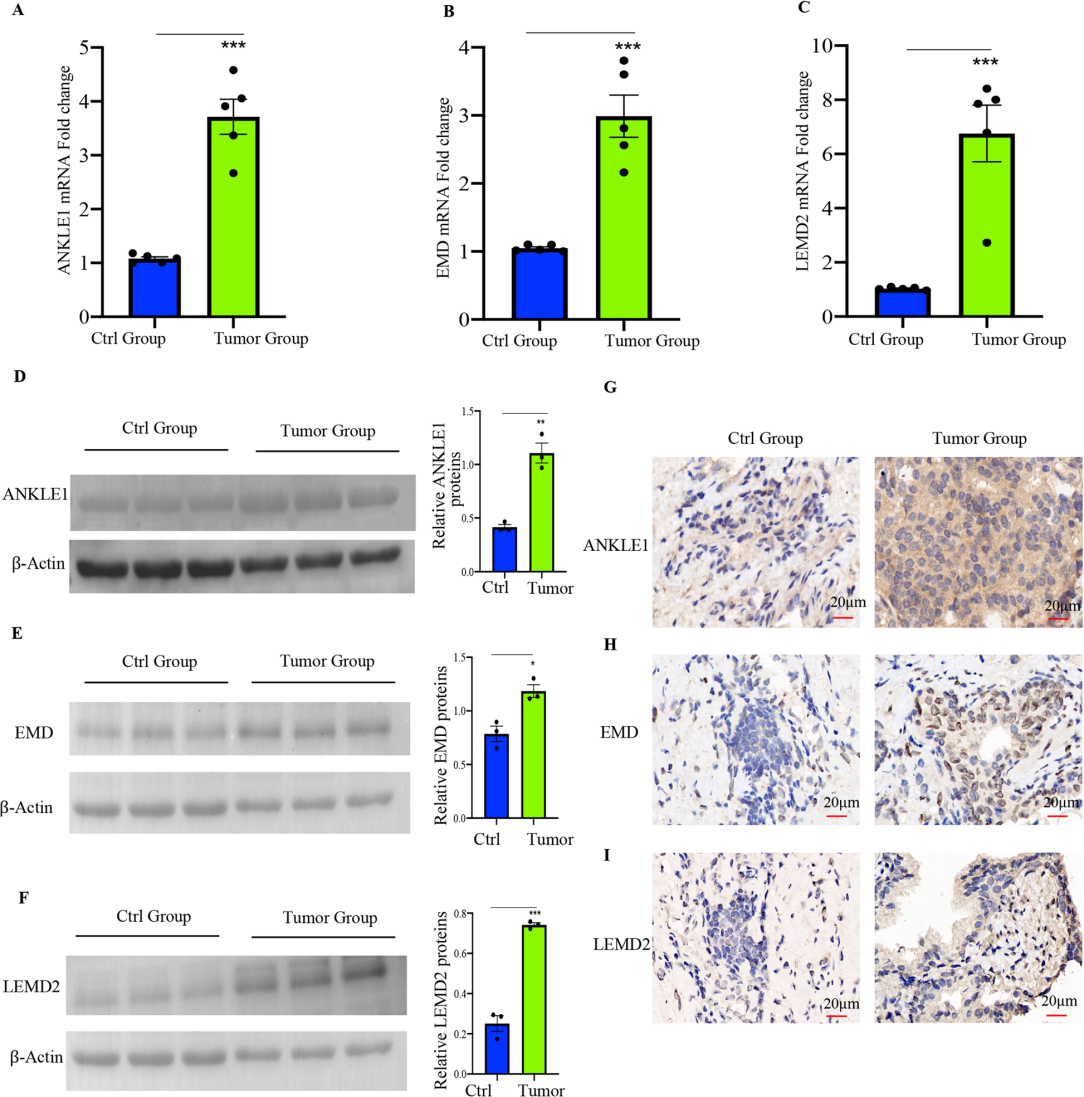
qPCR, WB, and IHC validation of ANKLE1, EMD, and LEMD2 in prostate cancer. **A**–**C** The mRNA levels of ANKLE1, EMD, and LEMD2 in human prostate tumor specimens were validated by qPCR. **D**–**I** The protein levels of ANKLE1, EMD, and LEMD2 in human prostate tumor specimens were validated by WB and IHC. The data (means ± SEM) shown (**A**–**C**, *n* = 5; **D**–**I**, *n* = 3) were representative of three separate experiments. Compared with the indicated group, *p < 0.05, **p < 0.01, ***p < 0.001

Furthermore, we also validated the mRNA and protein expression levels of ANKLE1, EMD, and LEMD2 in human prostate tumor specimens by qPCR, WB, and IHC (Fig. 3). Consistent with our predicted computational results, the mRNA and protein expression levels of ANKLE1, EMD, and LEMD2 were markedly increased in the PRAD group.

### Correlation of ANKLE1, EMD, and LEMD2 expressions with clinical characteristics of PRAD patients

Then, we investigated the association of ANKLE1, EMD, and LEMD2 expressions with different clinical characteristics of PRAD using the survival package of the R project (Additional file 1: Table S1). High ANKLE1 level was associated with poorer OS and RFI in white populations (OS: HR = 5.04, *p* < 0.05; PFI: HR = 3.06, *p* < 0.001) in PRAD. Similarly, upregulated level of EMD was correlated with worse prognostic outcomes in white populations (OS: HR = 4.28, *p* < 0.05; PFI: HR = 2.96, *p* < 0.001) in PRAD. The increased expression of LEMD2 was correlated with worse prognostic outcomes in white populations (PFI: HR = 4.47, *p* < 0.001) in PRAD. These results illustrated the prognostic value of the ANKLE1, EMD, and LEMD2 mRNA levels, following their clinical characteristics, particularly in the white populations of PRAD patients.

### The co-expression genes with ANKLE1, EMD, and LEMD2 in PRAD

**Fig. 4:**
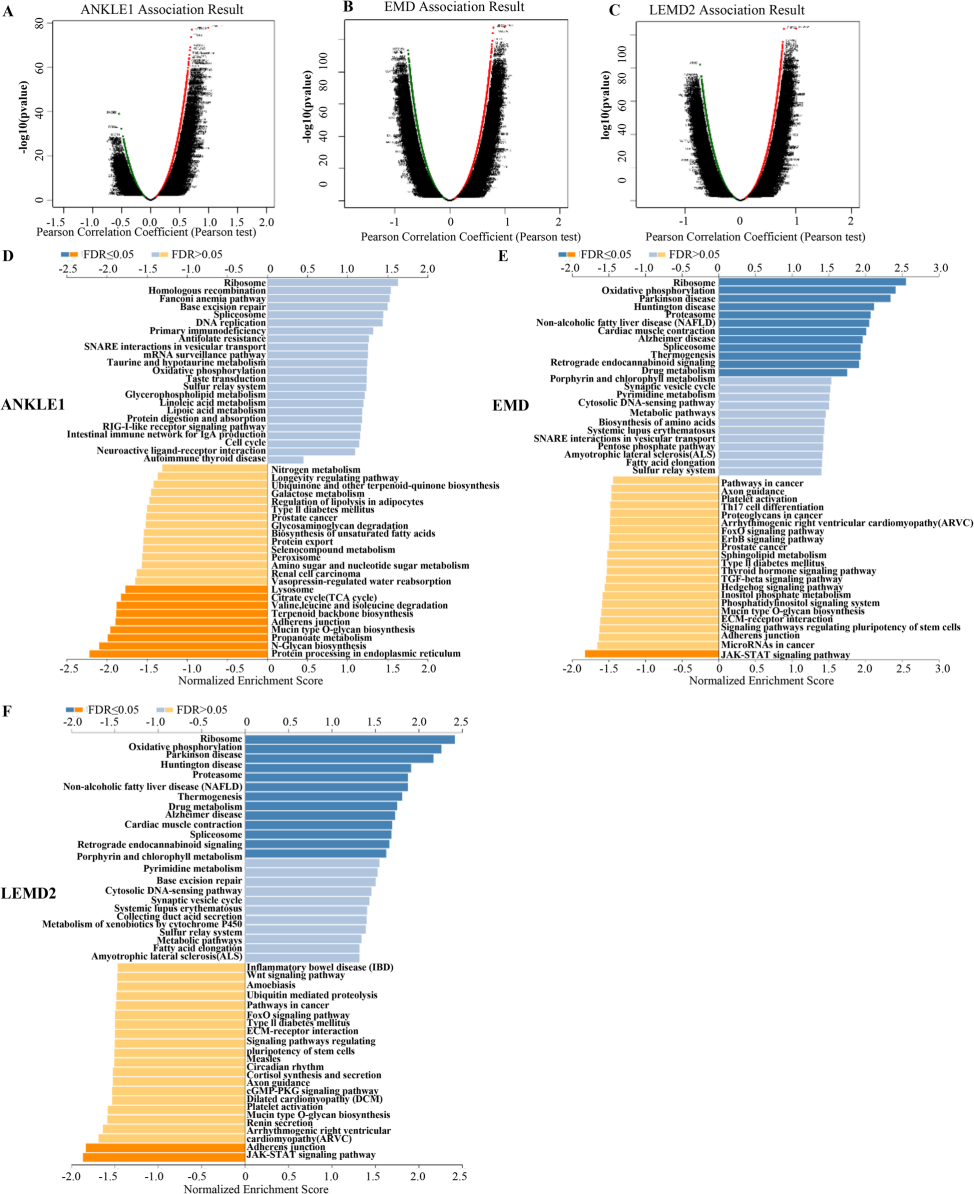
The co‐expression genes with ANKLE1, EMD, and LEMD2 in prostate adenocarcinoma from the LinkedOmics database. **A**–**C** The significantly associated genes with ANKLE1, EMD, and LEMD2 were distinguished by the Pearson test in the prostate adenocarcinoma cohort. **D**–**F** KEGG pathways of ANKLE1, EMD, and LEMD2 in prostate adenocarcinoma cohort

To gain knowledge of the ANKLE1, EMD, and LEMD2 biological function in PRAD, the LinkFinder module in the LinkedOmics web portal was employed to check the co-expression pattern of ANKLE1, EMD, and LEMD2 in TCGA-PRAD. As plotted in Fig. 4A, it showed that 7264 genes (dark red dots) positively correlated with ANKLE1, and 4711 genes (dark green dots) negatively correlated. Figure 4B showed that 6878 genes (dark red dots) positively correlated with EMD, and 7480 genes (dark green dots) negatively correlated. Similarly, in Fig. 4C, there were 7252 genes (dark red dots) positively correlated with LEMD2, and 7306 genes (dark green dots) negatively correlated. The heat maps of the top 50 genes positively and negatively associated with ANKLE1, EMD, and LEMD2 were shown in Additional file 1: Figs. S2A-S2B, S3A-S3B, S4A-S4B, respectively. It is worth noting that the top 50 positive genes of ANKLE1, EMD, and LEMD2 highly owned probability of becoming high-risk markers in PRAD (Additional file 1: Figs. S2C, S3C, S4C).

GO term annotation indicated that co-expressed genes of ANKLE1, EMD, and LEMD2 join mainly in oxidoreductase activity, acting on NADPH and mitochondrial protein complex (Additional file 1: Figs. S2D-S2F, S3D-S3F, S4D-S4F). KEGG pathway analysis showed mainly enrichment in the ribosome and oxidative phosphorylation (Fig. 4D–F).

These results showed a vast influence of ANKLE1, EMD, and LEMD2 expression networks on PRAD prognosis.

### ANKLE1, EMD, and LEMD2 expressions correlated with immune infiltration in PRAD

**Fig. 5:**
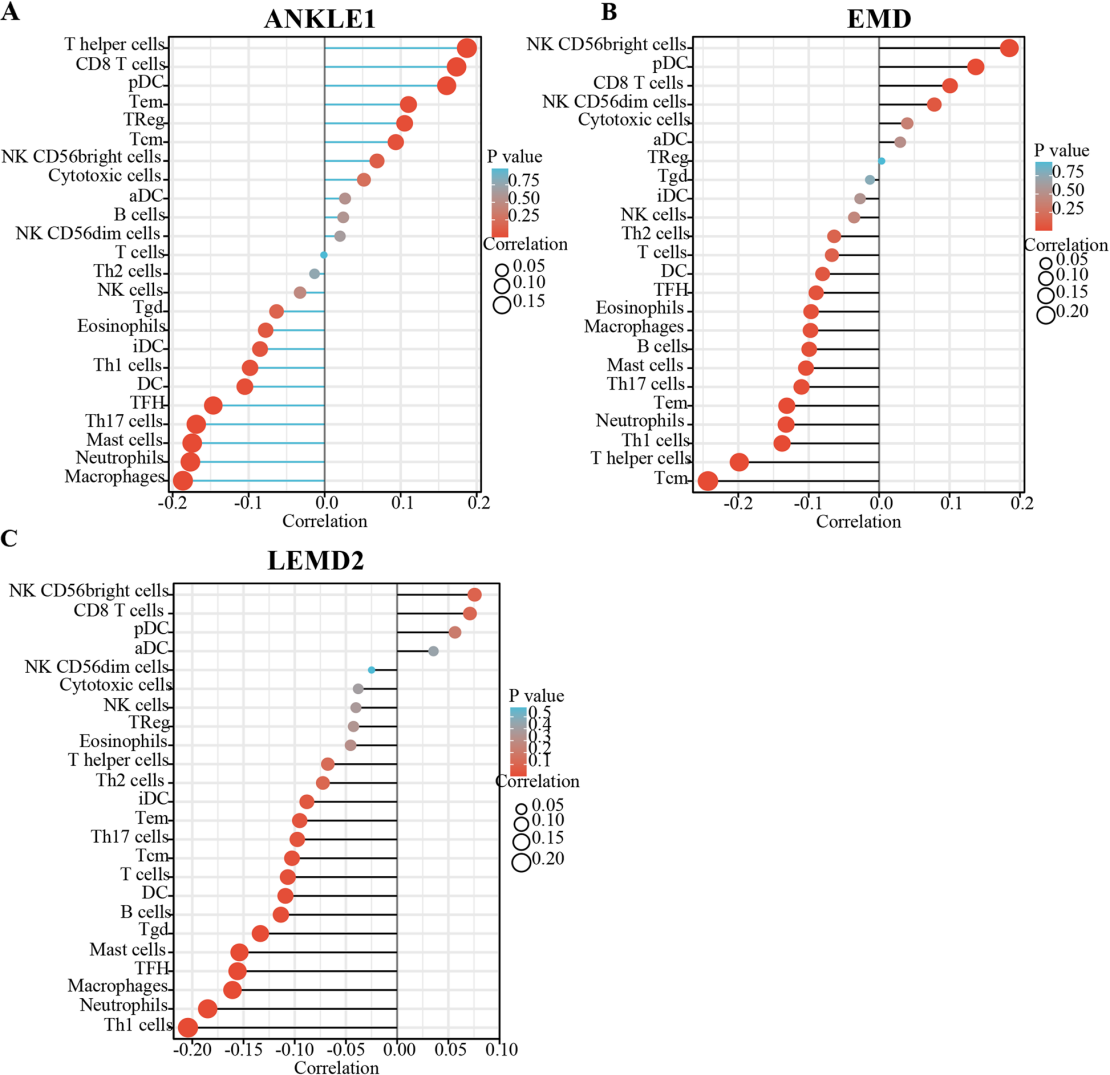
**A**–**C** The Correlation between ANKLE1, EMD, and LEMD2 expressions and infiltrating immune cells in prostate cancer

Tumor-infiltrating lymphocytes can independently be used to predict prognosis in cancers [29]. Here, we used the GSVA package of the R project to analyze the correlation of ANKLE1, EMD, and LEMD2 expressions with immune infiltration levels in PRAD. The results showed that ANKLE1 expression was significantly positively correlated with NK CD56^bright^ cells (*r* = 0.095, *p* = 0.034) and CD8 T cells (*r* = 0.187, *p* < 0.001) in PRAD (Fig. 5A), and ANKLE1 expression was significantly negatively correlated with macrophages (*r* = − 0.165, *p* < 0.001), neurophils (*r* = − 0.141, *p* = 0.002), mast cells (*r* = − 0.105, *p* = 0.02), and Th17 cells (*r* = − 0.171, *p* < 0.001) in PRAD (Fig. 5A). The expression of EMD was significantly positively correlated with NK CD56^bright^ cells (*r* = 0.185, *p* < 0.001), pDC (*r* = 0.137, *p* = 0.002), and CD8 T cells (*r* = 0.101, *p* = 0.025) in PRAD (Fig. 5B). The expression of EMD was significantly negatively correlated with B cells (*r* = -0.099, *p* = 0.027), macrophages (*r* = − 0.097, *p* = 0.03), mast cells (*r* = − 0.104, *p* = 0.02), neurophils (*r* = − 0.132, *p* = 0.003), Th1 cells (*r* = − 0.138, *p* = 0.002), and Th17 cells (*r* = − 0.11, *p* = 0.014) in PRAD (Fig. 5B). In addition, the expression of LEMD2 was not significantly correlated with NK CD56^bright^ cells (*r* = 0.076, *p* = 0.091) or CD8 T cells (*r* = 0.071, *p* = 0.113). However, LEMD2 expression was significantly negatively correlated with macrophages (*r* = − 0.161, *p* < 0.001), neutrophil cells (*r* = − 0.185, *p* = 0.015), mast cells (*r* = − 0.154, *p* < 0.001), dendritic cells (*r* = − 0.109, *p* < 0.001), B cells (*r* = − 0.114, *p* = 0.011), Th1 cells (*r* = − 0.204, *p* < 0.001), and Th17 cells (*r* = − 0.097, *p* = 0.03) in PRAD (Fig. 5C). In addition, we examined the prognostic value of ANKLE1, EMD, and LEMD2 levels and tumor-infiltrating resting NK cells in PRAD, using the Cox proportional hazard model by TIMER. The results showed that resting NK cells (*p* = 0.004) and ANKLE1 expression (*p* = 0.001) were significantly correlated with clinical prognosis in PRAD (Additional file 1: Table S2). Taken together, all of these results strongly implicated that ANKLE1, EMD, and LEMD2 could serve as major tumor immune infiltration regulators in PRAD.

### ANKLE1, EMD, and LEMD2 expressions correlated with immune stimulators and chemokines

**Fig. 6:**
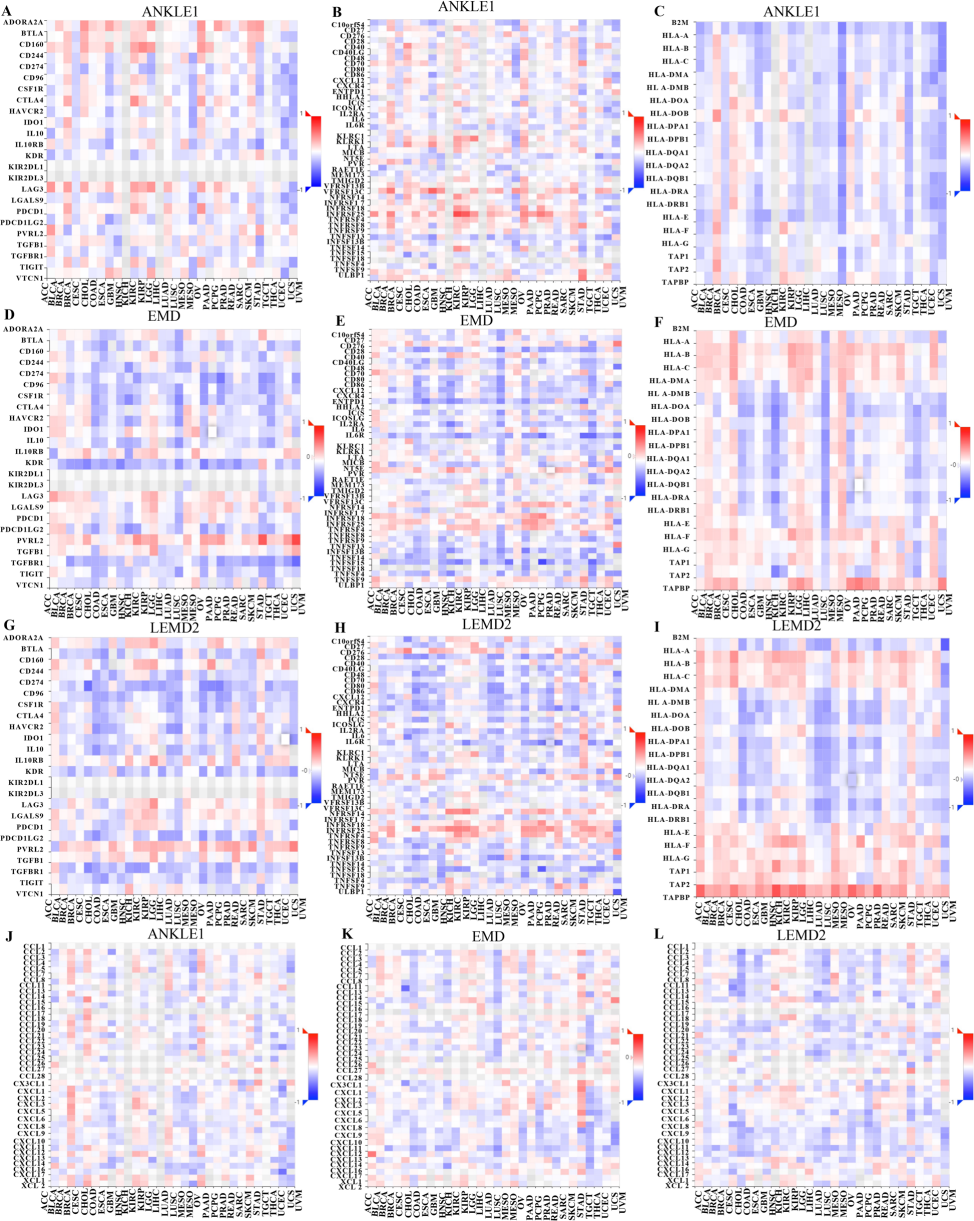
Associations of ANKLE1, EMD, and LEMD2 expressions with immunomodulators and chemokines from the TISIDB database. **A**–**I** Correlations between immunomodulators (including immune inhibitors, immunostimulators, and MHC molecules) and the expression levels of ANKLE1, EMD, and LEMD2. **J**–**L** Correlations between chemokines and the expression levels of ANKLE1, EMD, and LEMD2

We assessed the correlation of ANKLE1, EMD, and LEMD2 expressions and immune stimulators and chemokines in PRAD by exploring the TISIDB database. Our results showed that the ANKLE1, EMD, and LEMD2 levels in PRAD tissues were strongly associated with immune stimulators, including immune inhibitors, immunostimulators, and MHC molecules. Furthermore, the expression levels of ANKLE1, EMD, and LEMD2 correlated with chemokines (Fig. 6).

In addition, we found that the levels of ANKLE1, EMD, and LEMD2 were significantly correlated with various subtypes of T cell marker levels, including CD8^+^ T cell markers (CD8A, CD8B), general T cell markers (CD3D, CD3E, CD2), exhausted T cell marker (GZMB, LAG-3, PD-1), Th2 markers (GATA3), Th17 markers (STAT3), Treg markers (FOXP3, CCR8, TGF-b), Tfh marker (BCL6), neutrophils markers (ITGAM, CCR7), DC markers (CD1C, ITGAX), B cells markers (CD79A, CD19), and macrophages in PRAD (Additional file 1: Table S3) by TIMER web server. These findings revealed that ANKLE1, EMD, and LEMD2 were involved in regulating tumor immune infiltration in PRAD.

### Prognostic potential of ANKLE1, EMD, and LEMD2 expressions in PRAD based on immune cells

**Fig. 7:**
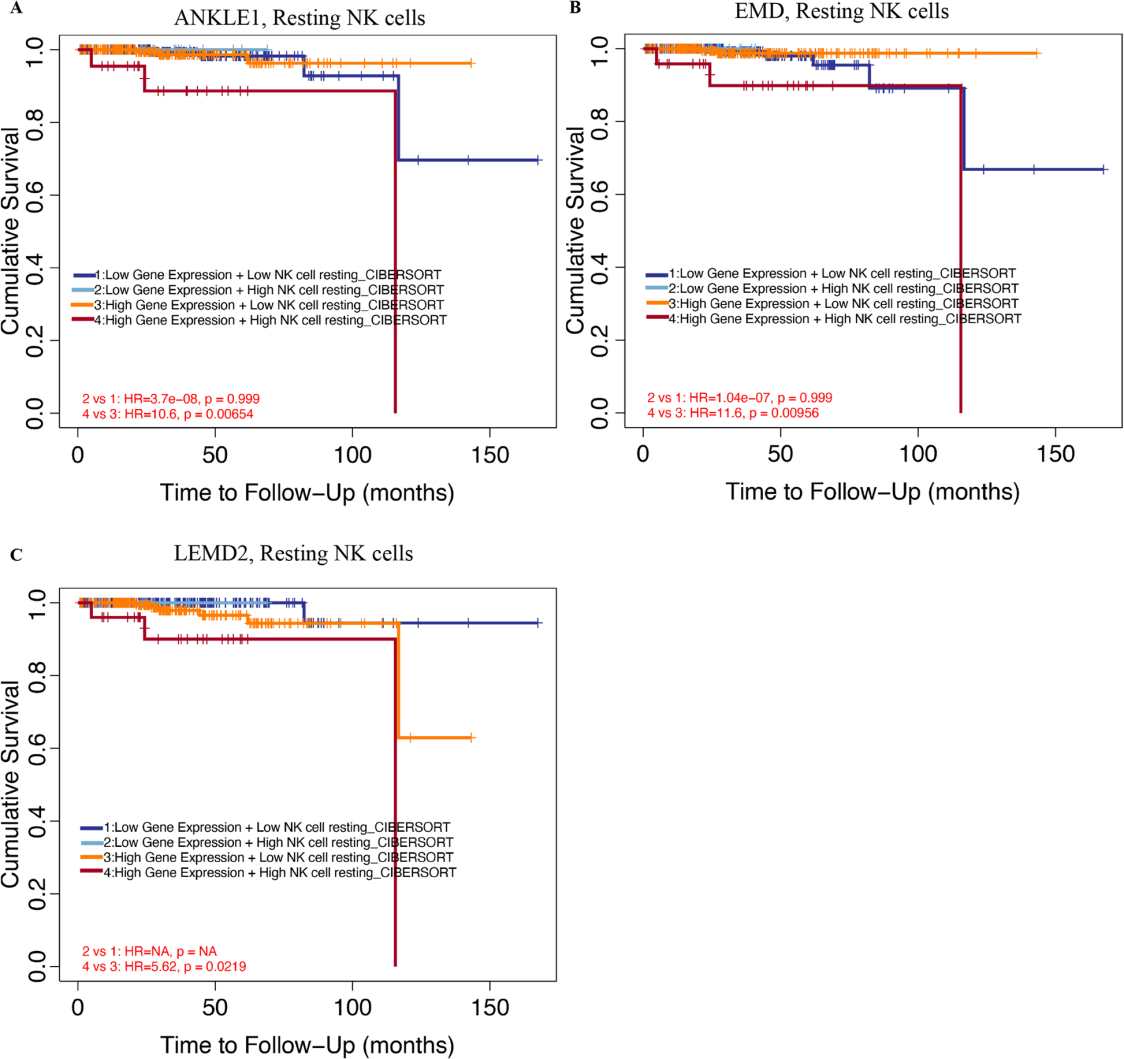
Comparison of KM survival curves of ANKLE1, EMD, and LEMD2 expressions based on immune cells. **A** High ANKLE1 levels enriched in resting NK cells had worse OS in PRAD. **B** High EMD levels enriched in resting NK cells had worse OS in PRAD. **C** High LEMD2 levels enriched in resting NK cells had worse OS in PRAD

This study showed that the levels of ANKLE1, EMD, and LEMD2 were associated with the immune infiltration of PRAD. Also, upregulated ANKLE1, EMD, and LEMD2 expressions have a worse prognosis in PRAD patients. Thus, we proposed a hypothesis that ANKLE1, EMD, and LEMD2 may partly affect PRAD patients’ prognosis through immune infiltration. Ten kinds of immune cells markedly infiltrated PRAD tissues, including resting NK cells, naïve B cells, resting dendritic cells, M2 macrophages, activated mast cells, neutrophils, M1 macrophages, monocytes, CD8^+^ T cells, and resting mast cells [30]. We performed Kaplan-Meier plotter analyses of ANKLE1, EMD, and LEMD2 levels in PRAD following the tumor-infiltrating immune cells mentioned above by using the TIMER database. We found that high ANKLE1, EMD, and LEMD2 levels in PRAD in enriched resting NK cells (*p* < 0.05) had a worse prognosis (Fig. 7). However, there were no significant differences between high and low ANKLE1, EMD, and LEMD2 expression groups in the overall survival in enriched naïve B cells, resting dendritic cells, M2 macrophages, activated mast cells, neutrophils, M1 macrophages, monocytes, CD8^+^ T cells, and resting mast cells (Additional file 1: Figs. S5, S6, and S7). The above analyses suggested that immune infiltration may, in part, affect the high ANKLE1, EMD, and LEMD2 expression prognosis of PRAD patients.

### The analysis of mutation, copy number variation, and methylation for ANKLE1, EMD, and LEMD2

**Fig. 8:**
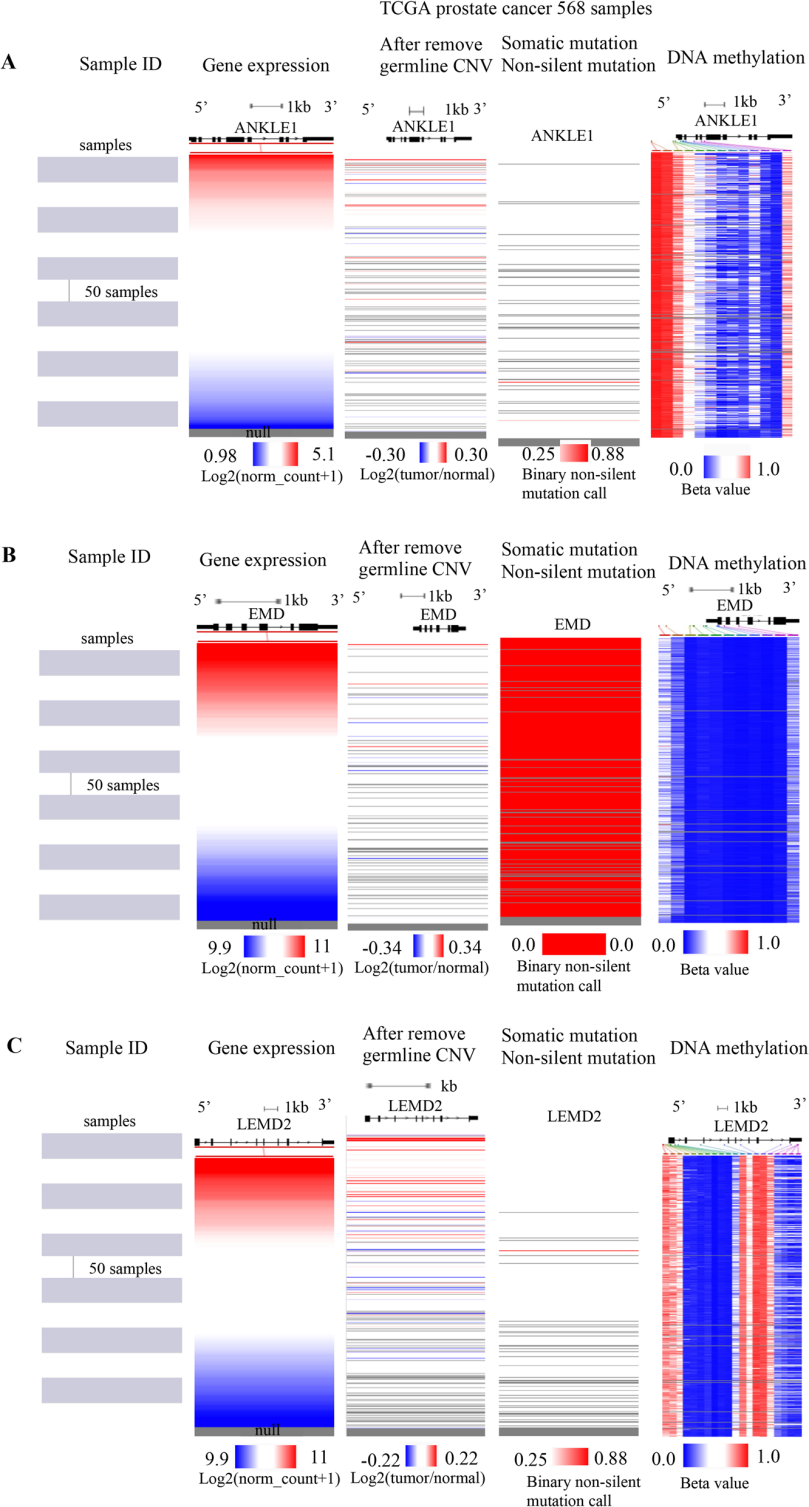
The analysis of mutation, CNV, and methylation for ANKLE1, EMD, and LEMD2 expressions in PRAD. **A**–**C** Heat map showing the correlations between ANKLE1, EMD, and LEMD2 mRNA levels and somatic mutations, CNV, and methylation in prostate cancer through the UCSC Xena database

The expression levels of ANKLE1, EMD, and LEMD2 were significantly elevated in PRAD. We assessed the cause of elevated ANKLE1, EMD, and LEMD2 levels. DNA methylation, gene mutation, and copy number variation (CNV) were critically involved in genetic and epigenetic regulation and were highly associated with the development of cancers. We verified the DNA methylation, gene mutation, and CNV levels of ANKLE1, EMD, and LEMD2 in PRAD via the UCSC Xena database. The results indicated that the expression of ANKLE1 mRNA correlated with DNA methylation, but not with CNV (*r* = 0.08451, *p* = 0.0611) and gene mutation (*r* = − 0.07674, *p* = 0.0884) in PRAD (Fig. 8A), and the expression of EMD mRNA was correlated with DNA methylation and positively correlated with CNV (*r* = 0.1583, *p* < 0.001), but not with a gene mutation in PRAD (Fig. 8B). In addition, the expression level of LEMD2 mRNA correlated with DNA methylation and positively associated with CNV (*r* = 0.2334, *p* < 0.0001), but not with a gene mutation in PRAD (Fig. 8C). Therefore, we suggested that DNA methylation might contribute to the elevated levels of ANKLE1 in PRAD, and CNV and DNA methylation may induce the increased levels of EMD and LEMD2 in PRAD.

## Discussion

PRAD is the most common malignant tumor in male cancer. Due to the lack of more sensitive and specific early diagnostic markers and specific drugs for PRAD treatment, the mortality rate of PRAD was very high if the tumor metastasis occurred. Then, biomarker analysis in PRAD has practical appeal, as reported in many studies revealing these biomarkers involved in the development of PRAD [31–35]. ANKLE1, EMD, and LEMD2 were members of the lamin-interacting proteins family. The other family members, LAP2 and LEMD1, were reported to be upregulated in various cancers. Hence, we comprehensively analyzed the expression and survival data of LEMs in PRAD patients from TCGA, LinkedOmics, and TISIDB databases. We found that all LEM expressions, except for LAP2, were markedly altered in PRAD compared to the normal samples. Among all LEMs, only the expressions of ANKLE1, EMD, and LEMD2 were correlated with advanced tumor stage and survival prognosis in PRAD. Survival analysis showed that high ANKLE1, EMD, and LEMD2 expressions were associated with poor OS and PFI in PRAD. In addition, we also further validated the mRNA and protein expression levels of ANKLE1, EMD, and LEMD2 in human prostate tumor specimens by qPCR, WB, and IHC. Consistent with the predicted computational results, the mRNA and protein expression levels of these genes were markedly increased in the PRAD group. These results indicated that ANKLE1, EMD, and LEMD2 were used as prognostic biomarkers for PRAD.

Then, we for the first time investigated the correlation between ANKLE1, EMD, and LEMD2 expression levels and immunotype markers in PRAD. The results implied that ANKLE1, EMD, and LEMD2 expressions were associated with immune infiltration in PRAD, especially in CD56^bright^ NK cells. For human NK cells, there are two subsets of NK cells, including CD56^bright^ NK cells and CD56^dim^ NK cells. In contrast, the CD56^bright^ subset expresses very little or no CD16 (CD56^bright^ CD16^low^), produces type I pro-inflammatory cytokines IFN gamma and tumor necrosis factor (TNF) alpha [36, 37], and has proangiogenic functions in non-small cell lung cancer patients [38, 39]. Moreover, induction of a proangiogenic and decidual-like CD56^bright^CD16^dim/-^CD9^+^CD49^+^ phenotype has also been observed in colorectal cancer patients [40]. These predicted correlations suggested the underlying mechanisms for ANKLE1, EMD, and LEMD2 regulation of CD56^bright^ NK cell function in PRAD. Hence, the poor prognosis of PRAD may be associated with its recruiting and regulating immune cells.

Through the Kaplan-Meier plotter analysis by the TIMER database, high expression levels of ANKLE1, EMD, and LEMD2 enriched in resting NK cell cohorts of PRAD had a worse prognosis. Since ANKLE1, EMD, and LEMD2 expression levels were mainly associated with CD56^bright^ NK cells, it is inferred that these genes enriched in resting CD56^bright^ NK cells of PRAD patients had a poor prognosis, which needs to be further validated by bioassays. In addition, co-expressed genes of ANKLE1, EMD, and LEMD2 were involved mainly in oxidoreductase activity. Oxidative phosphorylation-related genes were previously reported to be potential biomarkers of PRAD [41]. Then, it is necessary to investigate further the correlation between the oxidative phosphorylation of ANKLE1, EMD, and LEMD2 and immune cell infiltrating levels in PRAD in the future. Therefore, these results may explain that high expressions of ANKLE1, EMD, and LEMD2 partly affect the prognosis of PRAD patients through immune infiltration.

Genetic and epigenetic processes are very important in the regulation of gene expression [42]. This study further found that ANKLE1 expression was markedly correlated with DNA methylation and not with CNV or somatic variations. EMD and LEMD2 expressions were strongly correlated with DNA methylation and CNV, but not with gene mutation. DNA methylation is usually contributed to repress gene transcription and tumor progression [43, 44]. CNV and DNA copy number variation, including gene amplification, gain, loss, and deletion, affect the gene expression in the development of tumor growth [45]. Therefore, DNA methylation may cause ANKLE1 upregulated in PRAD, and DNA methylation and CNV may induce increased levels of EMD and LEMD2 in PRAD.

## Conclusions

In summary, the upregulated ANKLE1, EMD, and LEMD2 were strongly associated with clinicopathological features, poor prognosis, and immune cell infiltration. DNA methylation may attribute to ANKLE1 upregulated. DNA methylation and CNV may contribute to the increased levels of EMD and LEMD2 in PRAD. This study further found a new mechanism that ANKLE1, EMD, and LEMD2 expressions may affect the prognosis of PRAD through tumor immune infiltration. Hence, our study offers insights for further studies on tumor immunotherapy of PRAD. We also performed the validation bioassay for measurements of ANKLE1, EMD, and LEMD2 expression levels by qPCR, WB, and IHC. However, the current study is the preliminary part of a larger study. Undoubtedly, we will further study ANKLE1, EMD, and LEMD2 in PRAD to elaborate on the biological function of ANKLE1, EMD, and LEMD2 in the immune microenvironment and prognosis of PRAD patients.

## Supplementary Information

**Additional file 1: Figure S1.** The expression of LEM-domain proteins in PRAD. (A-C) The expression levels of ANKLE1, EMD and LEMD2 in different cancer tissues compared 42 to normal tissues in the TIMER database. (D-G) LEMD1, ANKLE2, TMPO and LEMD3 expression level in PRAD tissues compared to normal tissues (RNA-seq data from ATCG PRAD). The number of normal group is 52, the number of tumor group is 499. Compared with indicated group, * p<0.05, ** p<0.01, *** p<0.001, n.s.: no significant difference. **Figure S2.** The co-expression genes with ANKLE1 in prostate adenocarcinoma (PRAD) from the LinkedOmics database. (A-B) Top 50 genes positively and negatively related to ANKLE1 in PRAD showed, respectively, by heat maps. Red represents positively linked genes and blue represents negatively linked genes. (C) Survival map of the top genes positively and negatively associated with ANKLE1 54 in PRAD. (D-F) GO annotations of ANKLE1 in PRAD cohort. **Figure S3.** The co-expression genes with EMD in prostate adenocarcinoma (PRAD) from the LinkedOmics database. (A-B) Top 50 genes positively and negatively related to EMD in PRAD showed, respectively, by heat maps. Red represents positively linked genes and blue represents negatively linked genes. (C) Survival map of the top 20 genes positively and negatively associated with EMD in PRAD. 65 (D-F) GO annotations of EMD in PRAD cohort. **Figure S4.** The co-expression genes with LEMD2 in prostate adenocarcinoma (PRAD) from the LinkedOmics database. (A-B) Top 50 genes positively and negatively related to LEMD2 in PRAD showed, respectively, by heat maps. Red represents positively linked genes and blue represents negatively linked genes. (C) Survival map of the top 20 genes positively and negatively associated with LEMD2 in PRAD. 76 (D-F) GO annotations of LEMD2 in PRAD cohort. **Figure S5.** Comparison of Kaplan-Meier survival curves of the high and low expression of ANKLE1 in PRAD based on immune cells subgroups. (A–I) High ANKLE1 level enriched in Naïve B cells, resting dendritic cells, M2 macrophages, activated mast cells, neutrophils, M1 macrophage, monocytes, CD8 T cells and resting mast cells exhibited no significant differences in OS in PRAD. **Figure S6.** Comparison of Kaplan-Meier survival curves of the high and low expression of EMD in PRAD based on immune cells subgroups. (A–I) High EMD level enriched in Naïve B cells, resting dendritic cells, M2 macrophages, activated mast cells, neutrophils, M1 macrophage, monocytes, CD8 T cells and resting mast cells exhibited no significant differences in OS in PRAD. **Figure S7.** Comparison of Kaplan-Meier survival curves of the high and low expression of LEMD2 in PRAD based on immune cells subgroups. (A–I) High LEMD2 level enriched in Naïve B cells, resting dendritic cells, M2 macrophages, activated mast cells, neutrophils, M1 macrophage, monocytes, CD8 T cells and resting mast cells exhibited no significant differences in OS in PRAD. **Figure S8.** Raw data for western blot shown in Fig. 3D–F. **Table S1.** Correlation of ANKLE1, EMD and LEMD2 mRNA expression and prognosis in PRAD with different clinicopathological factors by R project. **Table S2.** The cox proportional hazard model of ANKLE1/135 EMD/LEMD2 and resting NK cells in PRAD (TIMER). **Table S3.** Correlation analysis between ANKLE1/155 EMD/LEMD2 and related gene markers of immune cells in prostate cancer samples (n=498) in TIMER 2.0.

## Data Availability

RNA-seq data can be obtained from TCGA (https://portal.gdc.cancer.gov/) PRAD. Data of this study are available upon request from the authors.

## Abbreviations

LEMs: Mammalian LEM-domain proteins
PRAD: Prostate adenocarcinoma
LAP2: Lamina-associated polypeptide 2
EMD: Emerin
BAF: Barrier-to-autointegration factor
CNV: Copy number variation
TNF: Tumor necrosis factor
HRs: Hazard ratios
CIs: Confidence intervals
GO_BP: Gene Ontology biological process
GO_CC: Gene Ontology cellular component
GO_MF: Gene Ontology molecular function
KEGG: Kyoto Encyclopedia of Genes and Genomes
GSEA: Gene set enrichment analysis
aDC: Activated DC
iDC: Immature DC
pDC: Plasmacytoid DC
Tcm: T central memory
Tem: T effector memory
Tfh: T follicular helper
Tgd: T gamma delta
