# Supplementary material for: Collective analysis of the expression and prognosis for LEM-domain proteins in prostate cancer
Source: World J Surg Oncol. 2022 Jun 2;20:174. doi: 10.1186/s12957-022-02640-z (PMC9161513; doi:10.1186/s12957-022-02640-z)
Supplement: Supplementary file 1 — Additional file 1: Figure S1. The expression of LEM-domain proteins in PRAD. (A-C) The expression levels of ANKLE1, EMD and LEMD2 in different cancer tissues compared 42 to normal tissues in the TIMER database. (D-G) LEMD1, ANKLE2, TMPO and LEMD3 expression level in PRAD tissues compared to normal tissues (RNA-seq data from ATCG PRAD). The number of normal group is 52, the number of tumor group is 499. Compared with indicated group, * p<0.05, ** p<0.01, *** p<0.001, n.s.: no significant difference. Figure S2. The co-expression genes with ANKLE1 in prostate adenocarcinoma (PRAD) from the LinkedOmics database. (A-B) Top 50 genes positively and negatively related to ANKLE1 in PRAD showed, respectively, by heat maps. Red represents positively linked genes and blue represents negatively linked genes. (C) Survival map of the top genes positively and negatively associated with ANKLE1 54 in PRAD. (D-F) GO annotations of ANKLE1 in PRAD cohort. Figure S3. The co-expression genes with EMD in prostate adenocarcinoma (PRAD) from the LinkedOmics database. (A-B) Top 50 genes positively and negatively related to EMD in PRAD showed, respectively, by heat maps. Red represents positively linked genes and blue represents negatively linked genes. (C) Survival map of the top 20 genes positively and negatively associated with EMD in PRAD. 65 (D-F) GO annotations of EMD in PRAD cohort. Figure S4. The co-expression genes with LEMD2 in prostate adenocarcinoma (PRAD) from the LinkedOmics database. (A-B) Top 50 genes positively and negatively related to LEMD2 in PRAD showed, respectively, by heat maps. Red represents positively linked genes and blue represents negatively linked genes. (C) Survival map of the top 20 genes positively and negatively associated with LEMD2 in PRAD. 76 (D-F) GO annotations of LEMD2 in PRAD cohort. Figure S5. Comparison of Kaplan-Meier survival curves of the high and low expression of ANKLE1 in PRAD based on immune cells subgroups. (A–I) High ANKLE1 level enriched in [file 12957_2022_2640_MOESM1_ESM.pdf]

Collective Analysis of the expression and Prognosis for LEM-Domain Proteins in Prostate Cancer

Tianzhen He [1, †, *], Yulian Zhang [2, †], Xueyu Li [3, †], Caihong Liu [4], Guanqun Zhu [3], Xinbao Yin [3], Zongliang Zhang [3], Kai Zhao [3], Zhenlin Wang [3], Peng Zhao [5, 6, *], and Ke Wang [3, *]

1. Institute of special environmental medicine, Nantong University, Nantong, 226019, China

2. Department of Gynecology, the Affiliated Hospital of Qingdao University, Qingdao University, No.16 Jiangsu Road, Shinan District, Qingdao, Shandong Province, 266000, China

3. Department of Urology, the Affiliated Hospital of Qingdao University, Qingdao University, No.16 Jiangsu Road, Shinan District, Qingdao, Shandong Province, 266000, China

4. Qingdao West Coast New District Health Bureau, Western Administrative Office Center, No. 166 Shuangzhu Road, Huangdao District, Qingdao, Shandong Province, 266000, China

5. Faculty of Sport Science and Coaching, Universiti Pendidikan Sultan Idris, Tanjong Malim, Perak Darul Ridzuan, 35900, Malaysia

6. Athletics Department, Duke Kunshan University, Kunshan, Jiangsu Province, 215316, China

[†] These authors contributed equally to this work and share first authorship.

* Correspondence

Tianzhen He
E-mail: sailing198562@ntu.edu.cn Tel.: +86 18136506060

Ke Wang
E-mail: wangke@qdu.edu.cn Tel.: +86 18663818807

Peng Zhao
E-mail: peng.zhao@dukekunshan.edu.cn Tel.: +86 15995416268

1.1 Supplementary Figures

Figure S1

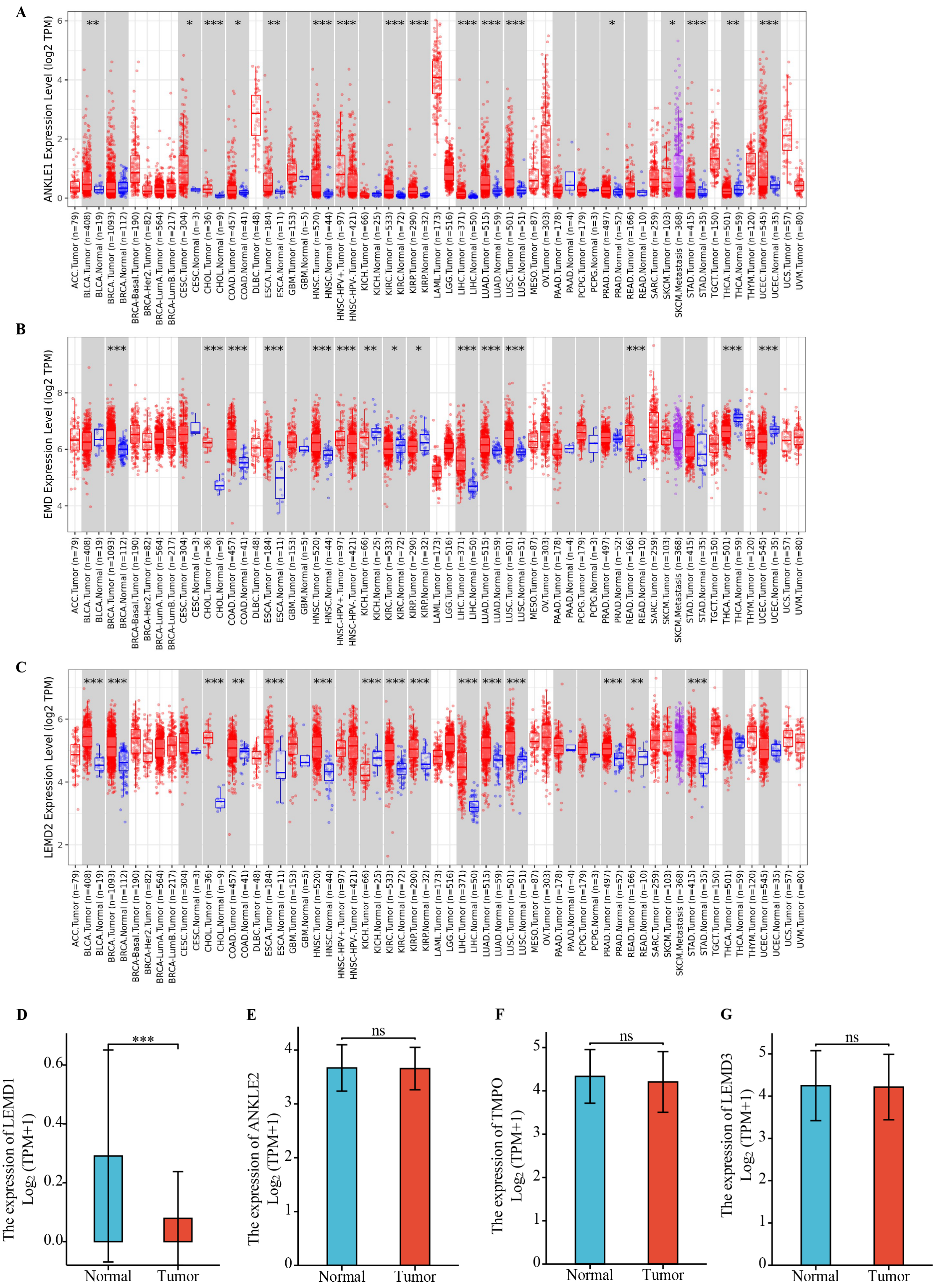

The expression of LEM-domain proteins in PRAD. (A-C) The expression levels of

ANKLE1, EMD and LEMD2 in different cancer tissues compared to normal tissues in the TIMER database. **(D-G)** LEMD1, ANKLE2, TMPO and LEMD3 expression level in PRAD tissues compared to normal tissues (RNA-seq data from ATCG PRAD). The number of normal group is 52, the number of tumor group is 499. Compared with indicated group, * $p<0.05$, ** $p<0.01$, *** $p<0.001$, n.s.: no significant difference.

Figure S2

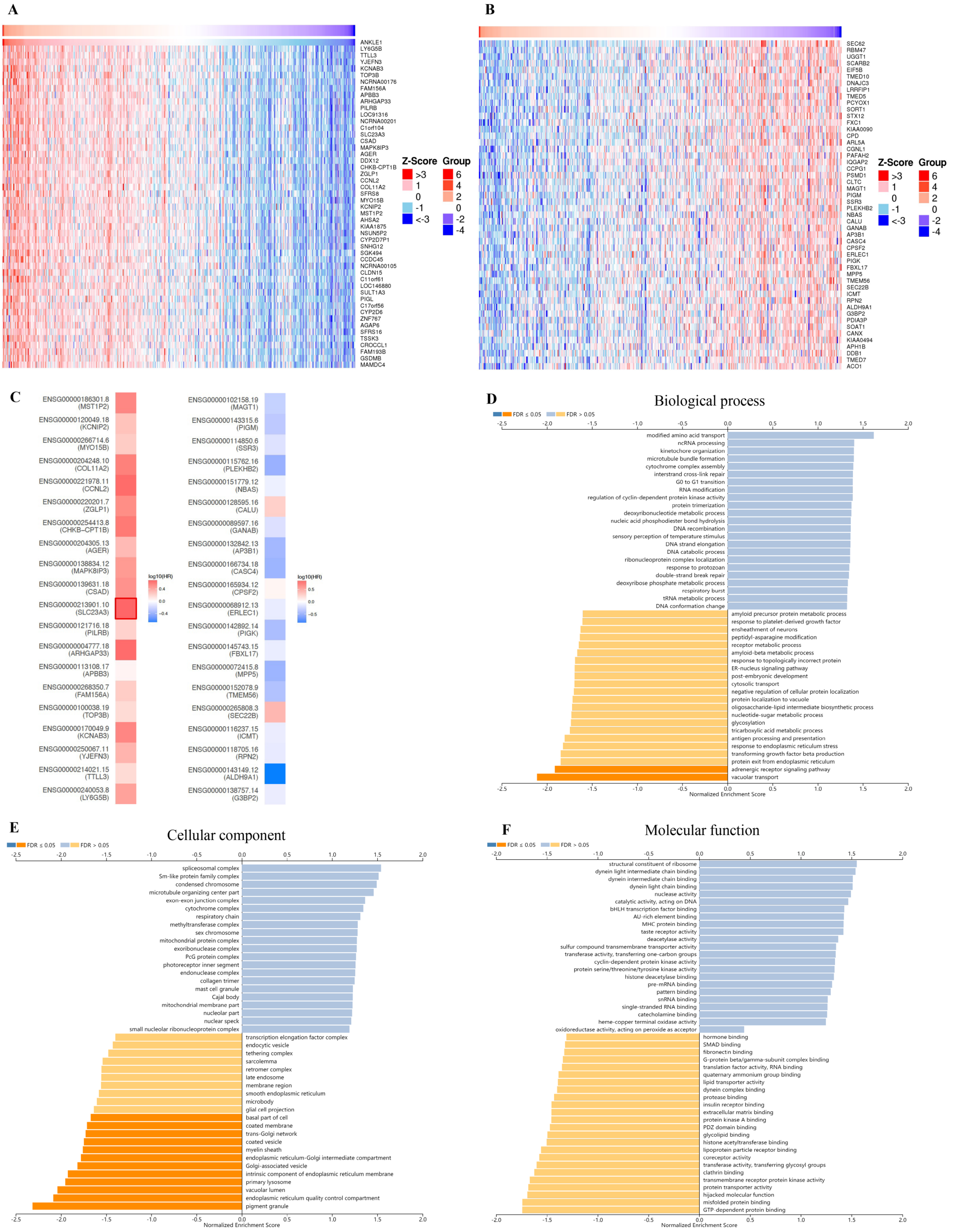

The co-expression genes with ANKLE1 in prostate adenocarcinoma (PRAD) from the LinkedOmics database. (A-B) Top 50 genes positively and negatively related to ANKLE1 in PRAD showed, respectively, by heat maps. Red represents positively linked genes and blue represents negatively linked genes. (**C**) Survival map of the top

20 genes positively and negatively associated with ANKLE1 in PRAD. (**D-F**) GO annotations of ANKLE1 in PRAD cohort.

Figure S3

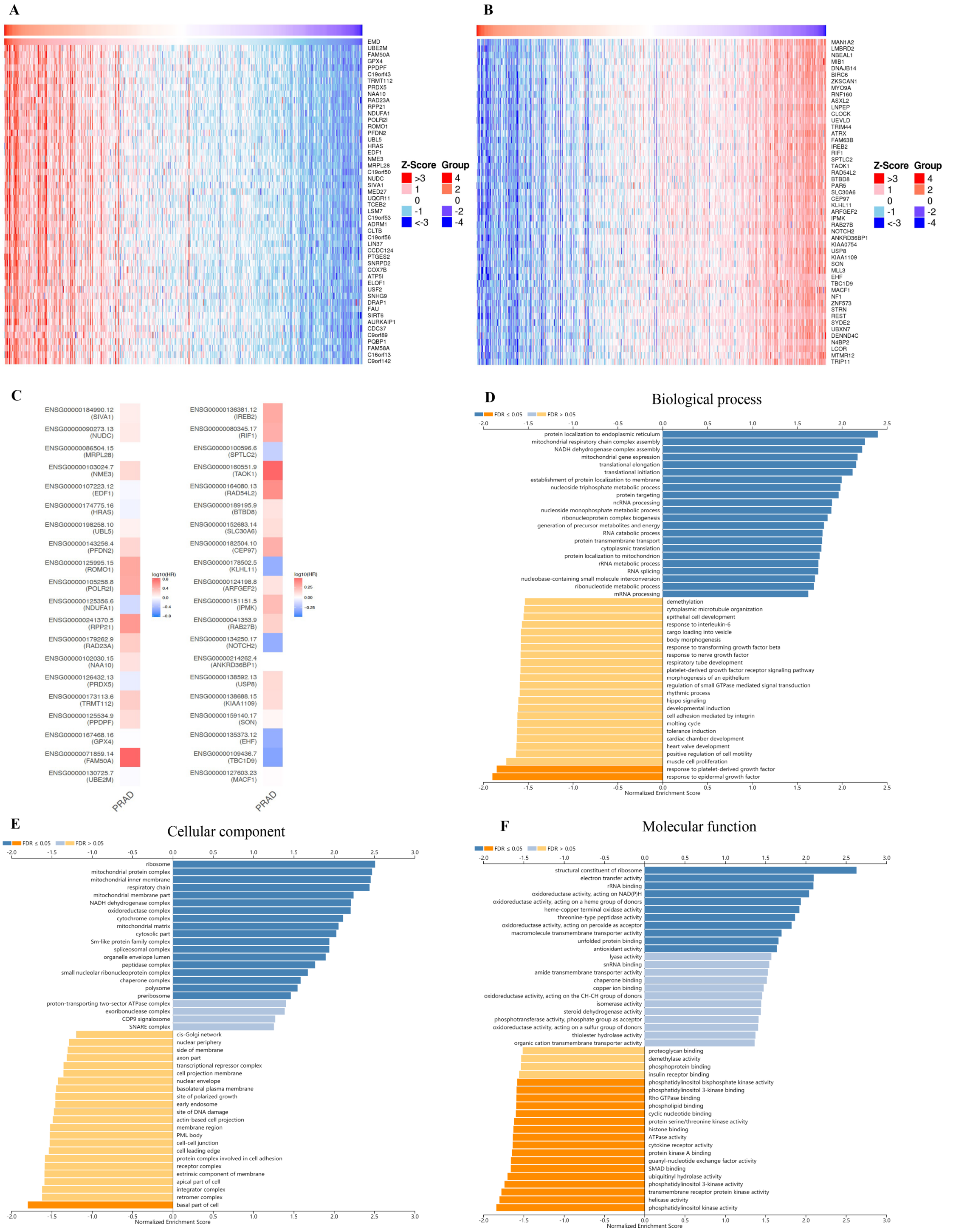

The co-expression genes with EMD in prostate adenocarcinoma (PRAD) from the LinkedOmics database. (A-B) Top 50 genes positively and negatively related to EMD in PRAD showed, respectively, by heat maps. Red represents positively linked genes and blue represents negatively linked genes. (**C**) Survival map of the top 20 genes

positively and negatively associated with EMD in PRAD. (**D-F**) GO annotations of EMD in PRAD cohort.

Figure S4

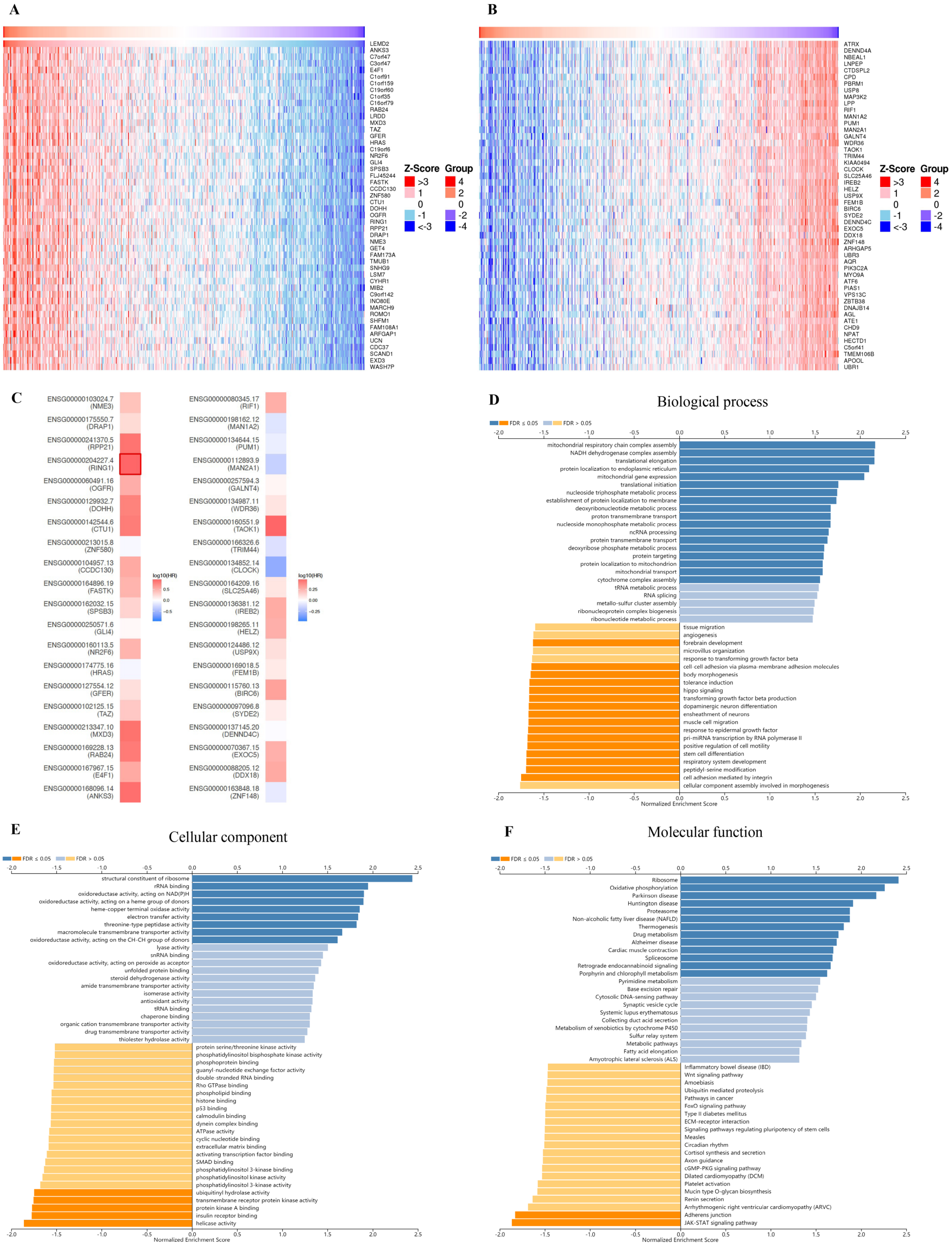

The co-expression genes with LEMD2 in prostate adenocarcinoma (PRAD) from the LinkedOmics database. (A-B) Top 50 genes positively and negatively related to LEMD2 in PRAD showed, respectively, by heat maps. Red represents positively linked genes and blue represents negatively linked genes. **(C)** Survival map of the top 20 genes

positively and negatively associated with LEMD2 in PRAD. (**D-F**) GO annotations of LEMD2 in PRAD cohort.

Figure S5

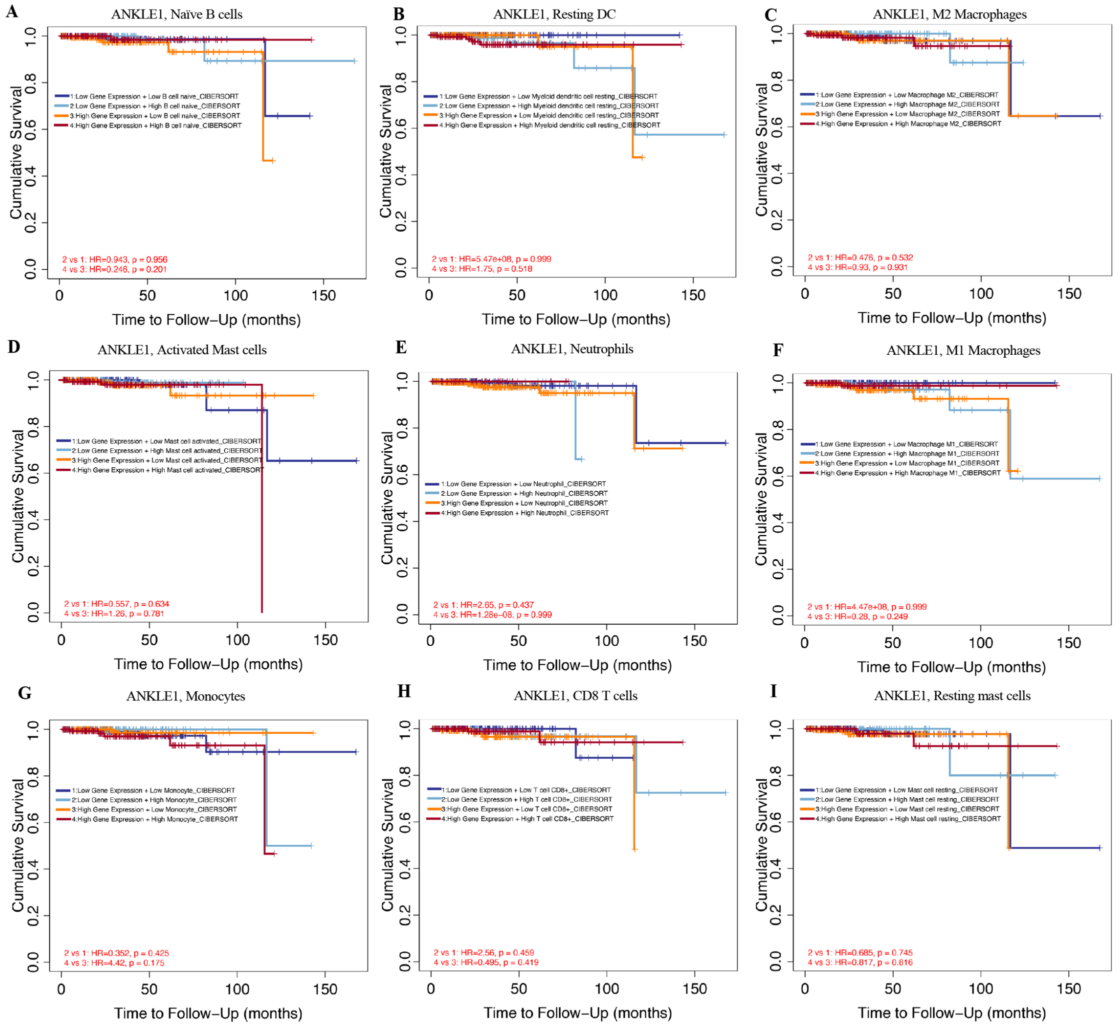

Comparison of Kaplan-Meier survival curves of the high and low expression of ANKLE1 in PRAD based on immune cells subgroups. (A–I) High ANKLE1 level enriched in Naïve B cells, resting dendritic cells, M2 macrophages, activated mast cells, neutrophils, M1 macrophage, monocytes, CD8 T cells and resting mast cells exhibited no significant differences in OS in PRAD.

Figure S6

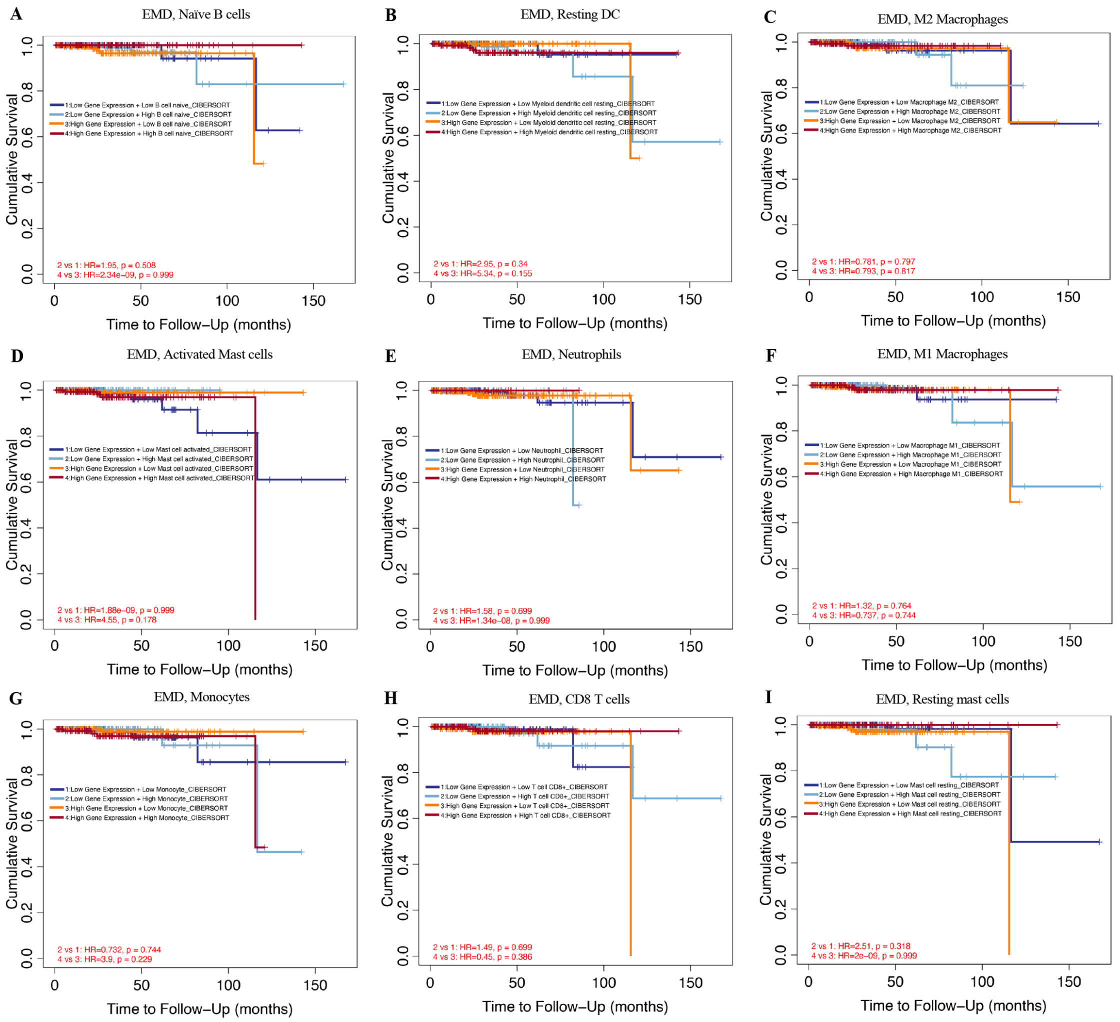

Comparison of Kaplan-Meier survival curves of the high and low expression of EMD in PRAD based on immune cells subgroups. (A–I) High EMD level enriched in Naïve B cells, resting dendritic cells, M2 macrophages, activated mast cells, neutrophils, M1 macrophage, monocytes, CD8 T cells and resting mast cells exhibited no significant differences in OS in PRAD.

Figure S7

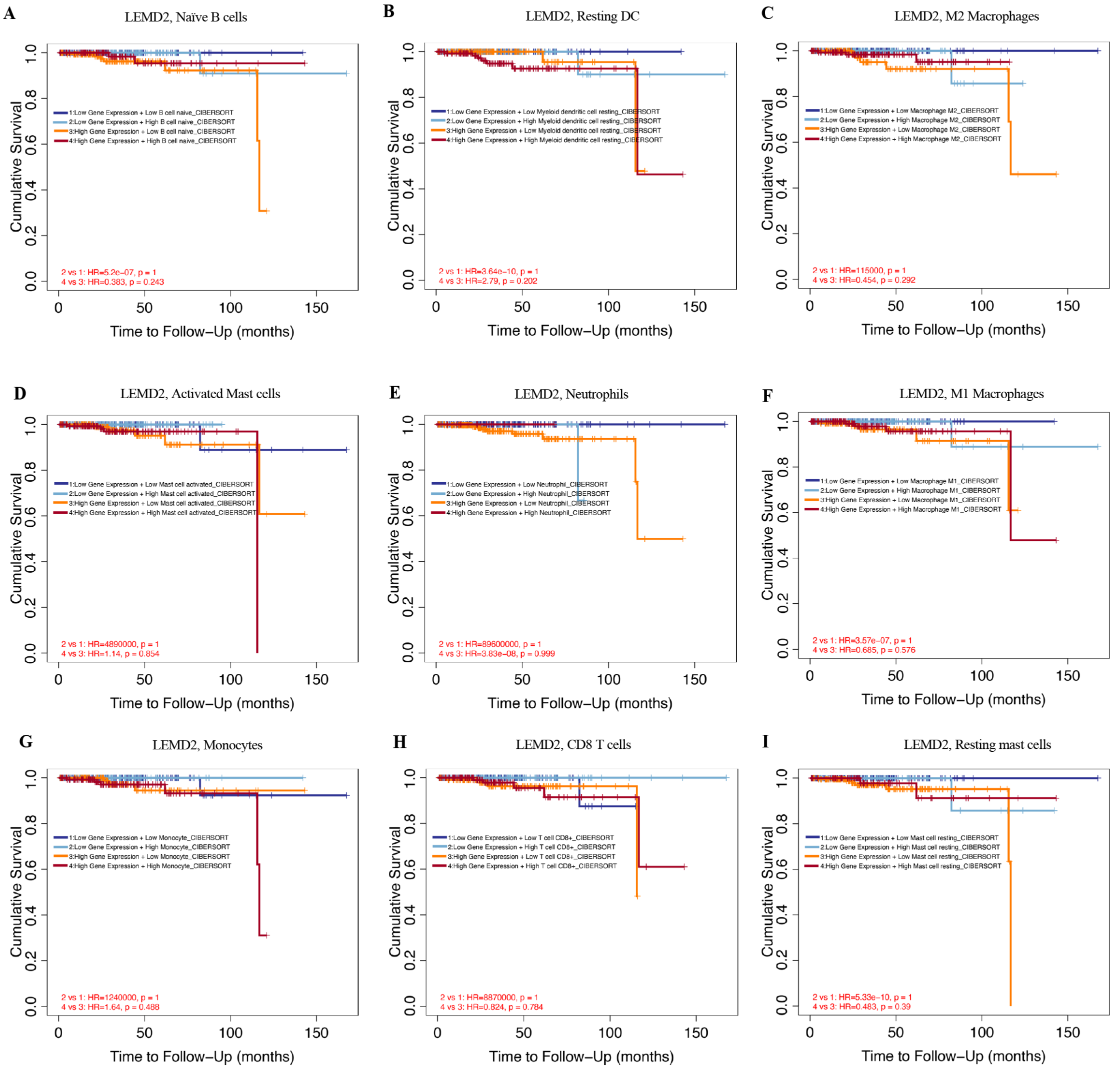

Comparison of Kaplan-Meier survival curves of the high and low expression of LEMD2 in PRAD based on immune cells subgroups. (A–I) High LEMD2 level enriched in Naïve B cells, resting dendritic cells, M2 macrophages, activated mast cells, neutrophils, M1 macrophage, monocytes, CD8 T cells and resting mast cells exhibited no significant differences in OS in PRAD.

Figure S8

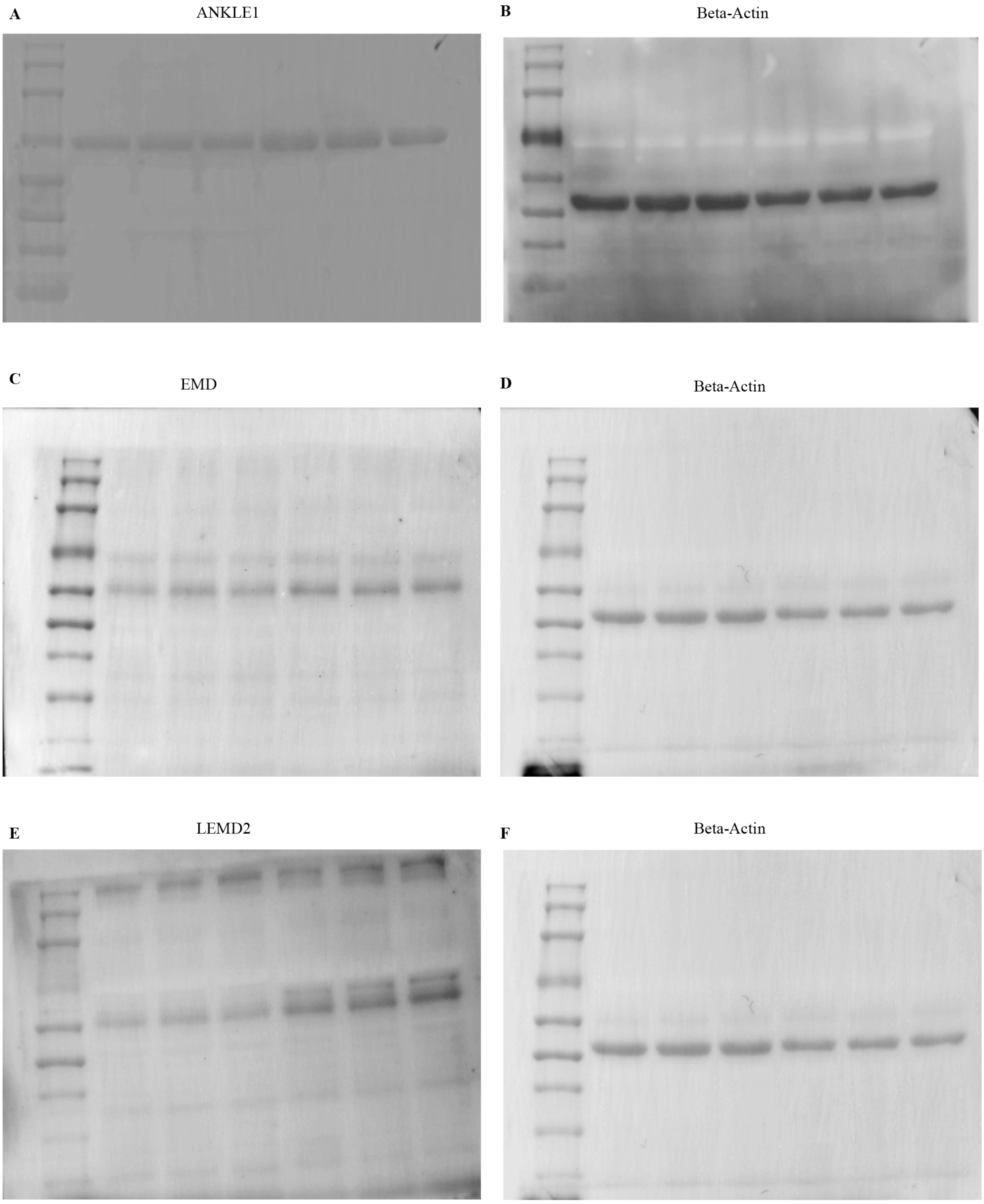

Raw data for western blot shown in Fig 3D-3F

1.2 Supplementary Tables

Table S1 Correlation of ANKLE1, EMD and LEMD2 mRNA expression and prognosis in PRAD with different clinicopathological factors by R project.

	ANKLE1				EMD				LEMD2			
Clinicopathological factors	OS		PFI		OS		PFI		OS		PFI	
	HR	p	HR	p	HR	p	HR	p	HR	p	HR	p
Age												
≤60	5.29	0.106	4.79	***	4.48	0.155	3.48	***	-	-	5.69	***
>60	3.1	0.13	3.55	**	3.56	0.085	2.76	***	-	-	3.82	***
Stage												
T2	-	-	4.3	**	-	-	0.46	0.134	-	-	7.71	**
T3	1.66	0.502	2.34	***	0.86	0.857	2.91	***	3.24	0.226	2.37	**
T4	1.95	0.222	2.77	0.231	0.28	0.18	3.09	0.188	0.71	0.648	3.69	0.114
N0	3.43	0.226	3.57	***	3.01	0.18	2.95	***	7.44	*	4.31	***
N1	2.02	0.557	1.73	0.213	3.38	0.291	1.99	0.093	0	0.118	1.76	0.287
Race												
Asian	2.87	0.109	-	-	0.54	0.386	-	-	2.87	0.109	-	-
Black or African American	1	1	4.91	*	0.14	0.083	2.09	0.338	0.14	0.083	5.13	*
White	5.04	*	3.06	***	4.28	*	2.96	***	-	-	4.47	***

* p<0.05, ** p<0.01, *** p<0.001

Table S2 The cox proportional hazard model of ANKLE1/EMD/LEMD2 and resting NK cells in PRAD (TIMER).

Model		coef	HR	95% CI_I	95% CI_u	p value
Surv(OS, EVENT) ~ `NK cell resting_CIBERSORT` + `ANKLE1`	Resting NK cells	48.235	8.87558E+20	4061175.298	1.93973E+35	0.004
	ANKLE1	3.131	22.901	3.439	152.512	0.001
Surv(OS, EVENT) ~ `NK cell resting_CIBERSORT` + `EMD`	Resting NK cells	45	3.56517E+19	2934.491	4.33139E+35	0.017
	EMD	1.444	4.238	0.568	31.62	0.159
Surv(OS, EVENT) ~ `NK cell resting_CIBERSORT` + `LEMD2`	Resting NK cells	41.043	6.67774E+17	857.855	5.1981E+32	0.019
	LEMD2	1.048	2.853	0.325	25.024	0.344

Table S3 Correlation analysis between ANKLE1/EMD/LEMD2 and related gene markers of immune cells in prostate cancer samples (n=498) in TIMER 2.0.

		ANKLE1				EMD				LEMD2			
Description	Gene markers	None		Purity		None		Purity		None		Purity	
		Cor	P	Cor	P	Cor	P	Cor	P	Cor	P	Cor	P
B cells	CD19	0.306	3.06E-05	0.154	1.58E-03	-0.049	2.72E-01	-0.022	6.61E-01	-0.012	7.98E-01	0.032	5.11E-01
	CD79A	0.09	4.36E-02	0.146	2.75E-03	-0.04	3.70E-01	-0.004	9.36E-01	-0.033	4.64E-01	0.007	8.80E-01
CD8$^+$ T cell	CD8A	0.151	6.98E-04	0.266	3.57E-08	0.043	3.35E-01	0.108	2.74E-02	-0.002	9.59E-01	0.084	8.85E-02
	CD8B	0.039	3.80E-01	0.09	6.62E-02	0.11	1.37E-02	0.134	6.12E-03	-0.049	2.79E-01	0.016	7.39E-01
Dendritic cell	ITGAX	0.333	2.18E-14	0.422	2.07E-19	0.092	3.91E-02	0.138	4.67E-03	0.259	4.34E-09	0.325	1.15E-11
	NRP1	0	9.91E-01	0.096	5.03E-02	0.32	2.41E-13	0.398	2.78E-17	0.154	5.50E-04	0.249	2.61E-07
	CD1C	0.12	7.38E-03	0.269	2.44E-08	0.002	9.72E-01	0.074	1.31E-01	0.002	9.68E-01	0.096	4.94E-02
	HLA-DPA1	0.081	7.19E-02	0.207	2.10E-05	0.084	2.85E-01	0.12	1.44E-02	0.027	5.53E-01	0.136	5.44E-03
	HLA-DRA	0.127	4.48E-03	0.247	3.32E-07	0.034	4.48E-01	0.106	3.02E-02	0.051	2.53E-01	0.143	3.36E-03
	HLA-DQB1	0.04	3.75E-01	0.108	2.80E-02	0.051	2.56E-01	0.063	2.01E-01	0.025	5.73E-01	0.103	3.63E-02
	HLA-DPB1	0.13	3.57E-03	0.203	3.09E-05	0.076	9.08E-02	0.093	5.92E-02	0.1	2.54E-02	0.159	1.15E-03
M1 Macrophage	PTGS2	-0.079	7.84E-02	0.006	9.04E-01	0.003	9.51E-01	0.056	2.58E-01	-0.123	5.95E-03	-0.043	3.86E-01
	IRF5	0.291	3.60E-11	0.358	5.20E-14	0.186	2.86E-05	0.208	1.89E-05	0.342	3.97E-15	0.373	3.79E-15
	NOS2	0.026	5.62E-01	0.085	8.32E-02	0.076	9.04E-02	0.129	8.34E-03	0.021	6.36E-01	0.104	3.43E-02
M2 Macrophage	MS4A4A	0.104	2.07E-02	0.193	7.25E-05	0.017	7.12E-01	0.108	2.79E-02	0.088	4.98E-02	0.18	2.26E-04
	VSIG4	0.074	9.96E-02	0.193	7.70E-05	-0.014	7.52E-01	0.083	9.21E-02	0.069	1.25E-01	0.17	4.89E-04
	CD163	0.062	1.65E-01	0.163	8.35E-04	-0.045	3.14E-01	0.049	3.16E-01	0.052	2.42-01	0.146	2.82E-03
Monocyte	CSF1R	0.117	9.23E-03	0.253	1.66E-07	-0.018	6.96E-01	0.05	3.11E-01	0.046	3.10E-01	0.145	3.13E-03
	CD86	0.138	2.04E-03	0.262	6.12E-08	0.012	7.97E-01	0.1	4.18E-02	0.067	1.36E-01	0.169	5.48E-04
Natural killer cell	KIR2DS4	0.059	1.87E-01	0.087	7.48E-02	0.055	2.17E-01	0.08	1.03E-01	0.026	5.57E-01	0.039	4.23E-01
	KIR3DL3	0.029	5.17E-01	0.063	2.03E-01	-0.053	2.34E-01	-0.076	1.23E-01	-0.013	7.71E-01	-0.022	6.48E-01
	KIR3DL2	-0.017	7.03E-01	0.019	6.92E-01	-0.058	1.93E-01	-0.044	3.70E-01	-0.077	8.81E-02	-0.037	4.47E-01
	KIR3DL1	0.017	7.05E-01	0.049	3.20E-01	0.023	6.07E-01	0.048	3.24E-01	-0.054	2.31E-01	-0.042	3.90E-01
	KIR2DL4	0.07	1.19E-01	0.111	2.31E-02	0.166	1.97E-04	0.186	1.35E-04	0.075	9.36E-02	0.107	2.86E-02
	KIR2DL3	-0.011	8.13E-01	-0.001	9.90E-01	0.086	5.37E-02	0.079	1.09E-01	0.025	5.75E-01	-0.003	9.49E-01
	KIR2DL1	0.074	1.00E-01	0.107	2.89E-02	0.109	1.45E-02	0.158	1.18E-03	0.072	1.10E-01	0.112	2.19E-02
Neutrophils	CCR7	0.095	3.36E-02	0.176	3.20E-04	-0.024	5.94E-01	0.017	7.28E-01	0.01	8.28E-01	0.071	1.48E-01
	ITGAM	0.184	3.76E-05	0.302	3.27E-10	-0.007	8.74E-01	0.073	1.37E-01	0.112	1.28E-02	0.213	1.15E-05
	CEACAM8	0.035	4.40E-01	0.034	4.83E-01	-0.068	1.31E-01	-0.074	1.33E-01	0.051	2.55E-01	0.048	3.28E-01
T cell (general)	CD3D	0.12	7.47E-03	0.188	1.16E-04	0.003	9.43E-01	0.028	5.72E-01	0.027	5.43E-01	0.083	9.03E-02
	CD3E	0.125	5.40E-03	0.218	7.31E-06	0.03	4.99E-01	0.077	1.16E-01	0.004	9.29E-01	0.073	1.40E-01
	CD2	0.136	2.42E-03	0.241	6.91E-07	-0.024	5.97E-01	0.023	6.43E-01	0.024	5.98E-01	0.098	4.50E-02
T cell exhaustion	CTLA4	0.226	3.33E-07	0.269	2.50E-08	0.001	9.89E-01	0.018	7.09E-01	0.162	2.94E-04	0.218	7.53E-06
	LAG3	0.234	1.34E-07	0.272	1.62E-08	0.077	8.54E-02	0.081	9.74E-02	0.128	4.24E-03	0.144	3.15E-03
	HAVCR2	0.17	1.35E-04	0.276	9.90E-09	0.05	2.61E-01	0.124	1.10E-02	0.129	3.82E-03	0.233	1.59E-06
	GZMB	0.05	2.69E-01	0.078	1.13E-01	0.097	3.13E-02	0.136	5.46E-03	0.041	3.64E-01	0.089	7.09E-02
	PDCD1	0.178	6.71E-05	0.214	1.02E-05	0.082	6.75E-02	0.085	8.30E-02	0.089	4.76E-02	0.111	2.32E-02
TAM	CCL2	0.057	2.03E-01	0.126	1.00E-02	0.073	1.02E-01	0.113	2.08E-02	-0.028	5.33E-01	0.012	8.14E-01
	IL10	0.091	4.28E-02	0.212	1.32E-05	-0.02	6.54E-01	0.049	3.22E-01	0.017	6.98E-01	0.12	1.40E-02
	CD68	0.144	1.31E-03	0.257	1.12E-07	0.001	9.88E-01	0.074	1.32E-01	0.094	3.55E-02	0.185	1.50E-04
Tfh	BCL6	0.112	1.22E-02	0.162	9.38E-04	0.138	1.98E-03	0.209	1.68E-05	0.077	8.44E-02	0.12	1.42E-02
	IL21	0.014	7.60E-01	0.028	5.69E-01	-0.038	3.93E-01	-0.046	3.46E-01	-0.016	7.24E-01	-0.015	7.59E-01
Th1	TBX21	0.259	4.72E-09	0.34	9.98E-13	0.101	2.43E-02	0.13	7.84E-03	0.178	6.24E-05	0.239	7.79E-07
	STAT4	0.267	1.42E-09	0.367	1.03E-14	0.07	1.17E-01	0.123	1.17E-02	0.121	7.07E-03	0.198	4.97E-05
	STAT1	0.025	5.81E-01	0.138	4.83E-03	0.031	4.92E-01	0.144	3.28E-03	0.061	1.76E-01	0.172	4.25E-04
	IFNG	0.144	1.25E-03	0.222	4.80E-06	-0.033	4.65E-01	0.007	8.81E-01	0.002	9.56E-01	0.063	1.99E-01
	IL13	0.145	1.21E-03	0.179	2.40E-04	0.168	1.72E-04	0.179	2.50E-04	0.115	1.00E-02	0.12	1.47E-02
Th2	GATA3	0.074	9.86E-02	0.19	9.40E-05	0.073	1.04E-01	0.116	1.82E-02	0.013	7.79E-01	0.085	8.51E-02
	STAT6	0.076	8.94E-02	0.166	6.70E-04	-0.093	3.76E-02	-0.069	1.59E-01	0.007	8.68E-01	0.076	1.19E-01
	STAT5A	0.138	2.08E-03	0.256	1.26E-07	0.03	5.09E-01	0.09	6.66E-02	0.047	2.98E-01	0.129	8.20E-03
Th17	STAT3	-0.071	1.15E-01	0.061	2.13E-01	0.072	1.10E-01	0.189	1.02E-04	0.019	6.75E-01	0.13	8.10E-03
	IL17A	-0.061	1.71E-01	0.041	4.00E-01	-0.115	1.05E-02	-0.05	3.12E-01	-0.088	4.99E-02	-0.014	7.73E-01
Treg	Foxp3	0.196	1.07E-05	0.282	4.59E-09	0.084	6.16E-02	0.113	2.13E-02	0.097	3.07E-02	0.147	2.72E-03
	CCR8	0.128	4.16E-03	0.24	7.07E-07	-0.057	2.02E-01	0.013	7.88E-01	0.015	7.45E-01	0.1	4.24E-02
	STAT5B	0.055	2.16E-01	0.198	4.65E-05	0.005	9.08E-01	0.109	2.69E-02	-0.012	7.94E-01	0.111	2.35E-02
	TGFB1	0.167	1.83E-04	0.229	2.49E-06	0.226	3.27E-07	0.265	3.87E-08	0.232	1.72E-07	0.283	4.11E-09
